# QTL analysis for sodium removal ability in rice leaf sheaths under salinity using an IR-44595/318 F_2_ population

**DOI:** 10.3389/fpls.2022.1002605

**Published:** 2022-10-11

**Authors:** Itsuki Goto, Sarin Neang, Ryuichi Kuroki, Vincent Pamugas Reyes, Kazuyuki Doi, Nicola Stephanie Skoulding, Mitsutaka Taniguchi, Akira Yamauchi, Shiro Mitsuya

**Affiliations:** ^1^ Laboratory of Plant Physiology and Morphology, Graduate School of Bioagricultural Sciences, Nagoya University, Nagoya, Japan; ^2^ Laboratory of Crop Stress Regulation, Graduate School of Bioagricultural Sciences, Nagoya University, Nagoya, Japan; ^3^ Ministory of Agriculture, Forestry and Fishery, Phnom Penh, Cambodia; ^4^ Laboratory of Information Sciences in Agricultural Lands, Graduate School of Bioagricultural Sciences, Nagoya University, Nagoya, Japan; ^5^ Laboratory of Organic Chemistry, Graduate School of Science, Nagoya University, Nagoya, Japan

**Keywords:** genotype-by-sequencing, leaf sheath, QTL, rice, salinity

## Abstract

Over-accumulation of salt in rice plants is an effect of salt stress which decreases growth and grain yield. Salt removal ability in leaf sheaths is a tolerance mechanism to decrease salt entry and accumulation in leaf blades and maintain photosynthesis under salinity. In this study, a QTL analysis of removal ability of sodium ions (Na^+^) in leaf sheaths and Na^+^ accumulation-related traits, was conducted using F_2_ population between two rice varieties, IR-44595 with superior Na^+^ removal ability, and 318 with contrasting Na^+^ removal ability in leaf sheaths under salinity. Suggestive QTLs for Na^+^ removal ability in leaf sheaths were found on chromosomes 4 and 11. The suggestive QTL on chromosome 11 overlapped with other significant QTLs for Na^+^ concentration in shoots, leaf blades and leaf sheaths, and Na^+^/K^+^ ratio in leaf blades. Correlation analysis indicated that Na^+^ removal ability in leaf sheaths is important in reducing Na^+^ accumulation in leaf blades. The varietal difference of Na^+^ removal ability in leaf sheaths at the whole plant level was greater at lower NaCl concentrations and became smaller as the treatment NaCl concentration increased. Although the Na^+^ removal ability in leaf sheath was comparable between IR-44595 and 318 under high salinity at the whole plant level, the younger leaves of IR-44595 still showed a higher Na^+^ sheath-blade ratio than 318, which implied the Na^+^ removal ability functions in the younger leaves in IR-44595 to reduce Na^+^ entry in young leaf blades even under high salinity.

## Introduction

Salt stress is one of the most influential abiotic stresses on rice productivity. To make the matters worse, recent climate change has resulted in increases in sea water levels, which has caused sea water intrusion into paddy fields especially in coastal areas. Therefore, to improve the rice productivity in such salt-prone areas, it is important to improve the salt tolerance in rice through molecular breeding using its quantitative trait loci (QTLs).

For salt tolerance of rice, protecting leaf blades, a major photosynthetic site in leaf organ, from toxic sodium ions (Na^+^) is most important to maintain photosynthesis and growth under salinity. Platten et al. (2013) have reported that salt tolerance is determined by Na^+^ accumulation level in leaf blades at the seedling stage in rice. Therefore, rice already has mechanisms to decrease Na^+^ entry and accumulation in leaf blades, namely salt blocking in roots using apoplastic barriers like Casparian strips, salt removal ability in roots, and salt removal ability in leaf sheaths (reviewed by Ismail and Horie, 2017). Of these traits, Na^+^ blocking and removal in roots has been well studied so far. Removal of Na^+^ in roots is mediated by a sodium transporter *OsHKT1;5* gene (Ren et al., 2005) and its QTLs (*Saltol* from Pokkali (Bonilla et al., 2002; Thomson et al., 2010) and *SKC1* from Nona Bokra (Lin et al., 2004)) have been used for molecular breeding of salt tolerant rice. Both *Saltol* and *SKC1* QTLs are localized on chromosome 1 (Negrão et al., 2011). Concerning salt blocking in roots, apoplastic barriers such as Casparian strips and suberin in root endodermis block salt entry *via* the apoplastic flow from outside into stele of rice roots (Krishnamurthy et al., 2011).

Rice has a salt (Na^+^ and Cl^-^) removal ability in leaf sheaths as well as roots. In rice leaf sheath, salt transported from roots *via* transpiration is unloaded (removed) from xylem vessels to the surrounding xylem parenchyma cells, then it is further transported to the fundamental parenchyma cells at the center (Neang et al., 2019). The salt ions carried in are actively sequestered in vacuoles. Therefore, leaf sheaths accumulate higher concentrations of Na^+^ and Cl^-^ compared with leaf blades and this contributes to decreasing their entry into leaf blades. The salt removal ability in leaf sheaths can be evaluated using a ratio of salt concentrations in leaf sheaths and leaf blades (James et al., 2006; James et al., 2011; Neang et al., 2019; Neang et al., 2020a; Neang et al., 2020b). Using this evaluation system, we performed a genome-wide association study and found significantly associated single nucleotide morphophisms (SNPs) with Na^+^ removal ability in leaf sheaths on chromosome 5 (Neang et al., 2020a). This GWAS analysis is the only report regarding the DNA markers for Na^+^ removal ability in leaf sheaths of rice, and more studies are necessary for identifying the causal genes of the Na^+^ removal ability in leaf sheaths, and for improving rice salt tolerance using the trait.

In this study, we performed a QTL analysis using F_2_ population between two rice varieties IR-44595 and 318 with contrasting Na^+^ removal ability in leaf sheaths. Na^+^ removal ability in leaf sheaths was evaluated using the Na^+^ sheath-blade ratio which was calculated by dividing Na^+^ concentration in leaf sheaths by the one in leaf blades (James et al., 2006; James et al., 2011; Neang et al., 2019; Neang et al., 2020a; Neang et al., 2020b). IR-44595 showed a high Na^+^ sheath-blade ratio whereas 318 showed a low ratio, indicating the superior Na^+^ removal ability of leaf sheaths of IR-44595 (Neang et al., 2020b). The main objective was to identify the QTL for Na^+^ removal ability in leaf sheaths. In addition, QTLs affecting Na^+^and potassium (K^+^) concentrations in shoots, leaf blades and leaf sheaths, and the Na^+^/K^+^ ratio were also analyzed. K^+^ is a counterpart ion of Na^+^ under salinity and decreases as more Na^+^ is absorbed in plants. Thus, the QTL for K^+^ concentration in plants and Na^+^/K^+^ ratio is also important for rice salt tolerance.

Moreover, in the preliminary experiment in this study, we found that the varietal difference in Na^+^ sheath-blade ratio was larger between IR-44595 and 318 under low salinity (25 mM NaCl treatment) but became smaller at the whole plant level as the treated NaCl concentration increased. It was speculated that the Na^+^ removal ability in leaf sheaths was comparable between two varieties under high salinity or the Na^+^ sheath-blade ratio at the whole plant level did not well represent the varietal difference of Na^+^ removal ability in leaf sheaths under high salinity. Therefore, we determined the Na^+^ removal ability in leaf sheaths under high salinity using younger (upper) leaves and older (lower) leaves separately. The hypothesis was, since rice preferentially accumulates salt in older leaves, salt entry is lesser in younger leaves so that younger leaf sheaths can efficiently remove Na^+^ and thus the Na^+^ sheath-blade ratio is higher in younger leaves in IR-44595 even under high salinity, but lower in older leaves.

## Materials and methods

### Plant materials and salt treatment

Two rice (*Oryza sativa* L.) varieties, IR-44595 (*indica*, IRGC_117759), and 318 (*tropical japonica*, IRGC_117629), were used in this study. The seeds were kindly provided by Genebank at IRRI. A preliminary experiment was performed to find the optimal NaCl concentration for NaCl treatment in QTL analysis. The seeds of two varieties were put into sodium hypochlorite solution with available chloride concentration at 5% for 5 min for surface sterilization, washed with tap water and rinsed with distilled water. The seeds were put on an wet paper towel in a petri dish and incubated in a growth chamber (12 h light, about 300 μmol m^-2^ s^-1^, 30°C; 12 h dark, 25°C) for germination for 3 days. Then the seedlings were transferred onto holes on a Styrofoam seedling float in distilled water-filled plastic containers. After 3 days, water was replaced with Yoshida’s hydroponic solution (Yoshida et al., 1976) and grown for two weeks. Then NaCl at a final concentration of 25, 50 or 100 mM was added to the hydroponic solution and the salt treatment was performed for 1 week. The pH was adjusted to between 5.0 to 5.2 daily using either KOH or HCl solution. The hydroponic solution was renewed every 3 days. The shoots were harvested and washed with distilled water to remove the contaminated salt on the surface from hydroponics. The shoots were separated into leaf blades and leaf sheaths and dried at 70°C for 3 days.

For soil culture, two germinated seeds of IR-44595 and 318 were sown in a plastic pot (159 mm diameter and 190 mm height) filled with 4.0 kg dried sandy loam soil thoroughly mixed with 2 g of ground complete fertilizer (N:P:K = 14:14:14). Plants were grown by irrigating tap water for 19 days. One seed was thinned 5 days after sowing. Then, NaCl at a final concentration of 0, 30 (as a low concentration) and 90 (as a high concentration) mM was added for the salt treatment. First, NaCl solution was added from the top and drained two times to substitute the soil solution in the pot with NaCl solution. Then tap water (for control) or NaCl solution was kept in pots at 1-2 cm above the soil surface and grown for further 3 weeks. Plant cultivation was conducted in a greenhouse at Nagoya University (lat. 35°9′10″N, long. 136°58′15″E). Shoots were harvested and washed with distilled water. After separating tillers, all leaves on the main stem (leaf age was 10 in IR-44595 and 8 in 318) were separated into leaf blades and leaf sheaths and dried at 70°C for 3 days. Leaves were divided into younger (leaf age 7-10 in IR-44595 and 6-8 in 318) and older leaves (leaf age 3-6 in IR-44595 and 3-5 in 318) and used for further measurement. The first and second leaves were etiolated under all treatment so excluded from the measurement.

For QTL analysis, a mapping population of 168 F_2_ lines was generated from a cross between IR-44595 and 318. Plant cultivation was conducted in a greenhouse at Nagoya University. All the F_2_ population were hydroponically grown as described above for 18 days and treated with NaCl by adding 30 mM NaCl to the hydroponics for one week. Then, 3rd to 7th fully developed leaves on the main stem were harvested, divided into leaf blades and leaf sheaths, and used for the measurement of Na^+^ and Cl^-^ concentrations as described above.

### Measurement of Na^+^ and K^+^ concentrations

Samples were used for the measurement of Na^+^ and K^+^ concentrations with Atomic Absorption spectrometry (iCE 3000, Thermo Scientific) in emission mode as described in Neang et al. (2019).

### Genotyping by sequencing and QTL mapping

For genotyping, DNA was extracted from tillers using a slightly modified method of Dellaporta et al. (1983). The modified point was that 10% (w/v) sodium dodecyl sulfate (SDS) was added to the extraction buffer. A GBS library was prepared according to Phung et al. (2019) and used for next generation sequencing on a HiSeq (Illumina Inc., San Diego, CA, USA) together with 20% spike in of PhiX (PhiX Control v3, Illumina). Informatics was conducted as described in Furuta et al. (2017) to obtain genotypes. 2221 single nucleotide polymorphism (SNP) markers were obtained. QTL analysis was based on interval mapping with the “scanone” function implemented in R/qtl (Broman et al., 2003). Significant QTLs were identified based on an empirical threshold determined by 1000 permutation tests (Churchill and Doerge, 1994). The estimated percent variance explained by the QTL is calculated from logarithm of the odds (LOD) values at the peaks of the QTL following Broman and Sen (2009):


$$\text{The estimated percent variance explained by the QTL}=\;1\;–\;10^{-\left(2/\text{n}\right)\text{*LOD}}$$

### Statistical analysis

Four replicates were used per treatment. Data were statistically analysed between two varieties under each treatment using Student’s *t*-test using R software (R version 4.0.5) (R Core Team, 2021). Correlations among traits in the F2 family at seedling stage in the presence of 30 mM NaCl in hydroponics imposed with one week were calculated using Spearman correlation method using R software.

## Results

### Varietal difference in Na^+^ removal ability in leaf sheaths

**Figure 1:**
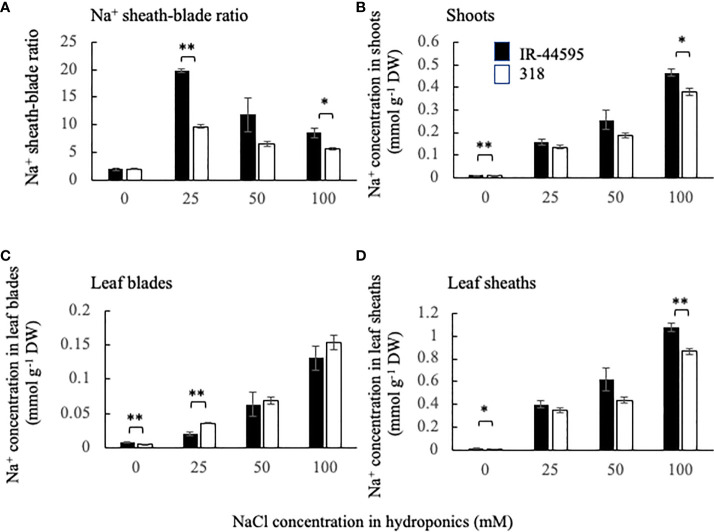
Varietal difference of Na^+^ sheath-blade ratio **(A)** and Na^+^ accumulation in shoots **(B)**, leaf blades **(C)**, and leaf sheaths **(D)**. Black and white bars represent IR-44595 and 318. Data are mean of four replications ± the standard error. * and ** indicate significant difference at P<0.05 and 0.1 between IR-44595 and 318 under each treatment (Student’s *t*-test).

To decide the concentration of NaCl treatment for QTL analysis, the sheath-blade ratio of Na^+^ concentration was determined under 0, 25, 50 and 100 mM NaCl treatment. IR-44595 showed a higher Na^+^ sheath-blade ratio compared with 318 under the treatment of 25 and 100 mM NaCl (**Figure 1A**). The varietal difference was biggest under 25 mM NaCl treatment (about 2 times) and became smaller with increasing salt treatment concentrations (1.5 times under 100 mM NaCl treatment). Under 25 mM NaCl treatment, Na^+^ concentration in the whole shoots was comparable between two varieties (**Figure 1B**) whereas IR-44595 showed lower Na^+^ concentration in leaf blades than 318 (**Figure 1C**). In addition, under 100 mM NaCl treatment, IR-44595 accumulated higher Na^+^ concentration in the shoots and leaf sheaths (**Figures 1B, D**). However, leaf blades showed comparable Na^+^ concentration between two varieties (**Figure 1C**). From these results, treatment of low NaCl concentration (25 mM NaCl in this preliminary experiment) was suggested for phenotyping in the following QTL analysis since the varietal difference of Na^+^ removal ability in leaf sheaths was larger between IR-44595 and 318. Therefore, 30 mM NaCl was used in the following QTL analysis as a low NaCl concentration.

### QTL for Na^+^ removal ability in leaf sheaths and related traits under salinity

**Figure 2:**
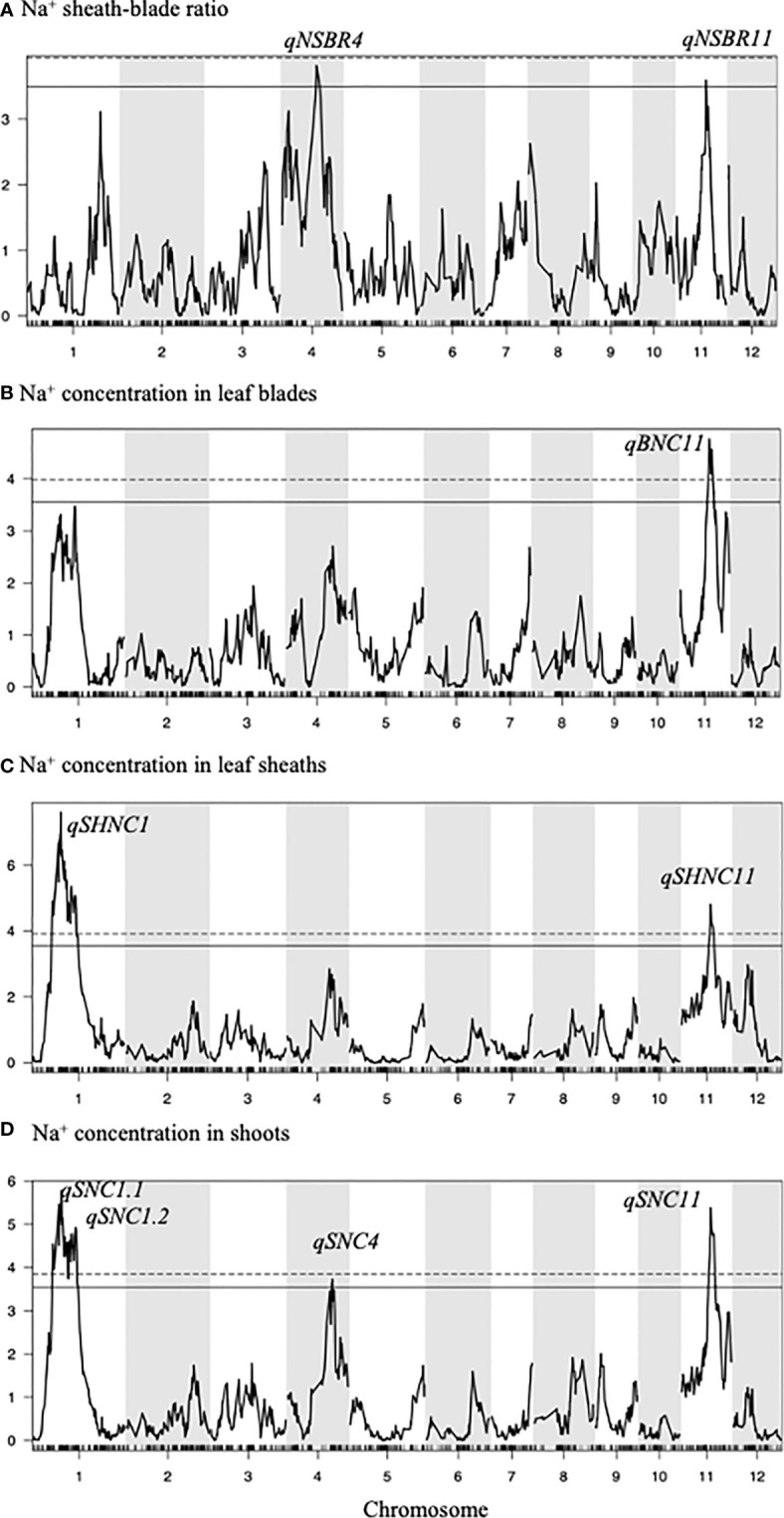
Interval mapping showing the QTLs. Trait is shown on each plot. Horizontal dotted and solid lines in all plots indicate am empirical threshold at the 5% and 10% level, respectively. For Na^+^ sheath-blade ratio, no significant QTL was detected but qNaSBR4 (LOD = 3.81) and qNaSBR11 (LOD = 3.59) were shown as a suggestive QTL (over the empirical threshold at the 10% level).

For QTL analysis, 2221 single nucleotide polymorphism (SNP) markers were obtained by GBS and used for interval mapping (**Figure 2**, **Table 1**, **Supplementary Table 1**, **Supplementary Figures 2, 3**).

**Table 1:** QTL detected in F_2_ generation for traits for Na^+^ sheath-blade ratio and salt tolerance-related traits from the cross of IR-44595 and 318 in the presence of 30 mM NaCl in hydroponics at the seedling stage based on interval mapping.

QTL	Chr^a^	Trait	Marker	Position (Mb)	LOD	AE^b^	PVE^c^ (%)
*qSNC1-1*	1	Shoot Na^+^ concentration	S01_10148605	10.1	5.78	-0.041	14.73
*qSNC1-2*	1	Shoot Na^+^ concentration	S01_22291976	22.3	4.97	-0.039	12.81
*qSHNC1*	1	Leaf sheath Na^+^ concentration	S01_10148605	10.1	7.60	-0.092	18.91
*qSNC4*	4	Shoot Na^+^ concentration	S04_27915871	27.9	3.72^d^	-0.030	9.75
*qNSBR4*	4	Na^+^ sheath-blade ratio	S04_24088589	24.1	3.81^d^	1.03	9.92
*qSNC11*	11	Shoot Na^+^ concentration	S11_19094522	19.1	5.38	0.043	13.79
*qBNC11*	11	Leaf blade Na^+^ concentration	S11_19375230	19.4	4.77	0.016	12.90
*qSHNC11*	11	Leaf sheath Na^+^ concentration	S11_19094522	19.1	4.80	0.081	12.40
*qBKNR11*	11	Leaf blade K^+^-Na^+^ ratio	S11_19375230	19.4	6.94	-4.23	17.61
*qNSBR11*	11	Na^+^ sheath-blade ratio	S11_19375230	19.4	3.59^d^	-1.03	9.37

a Chromosome.

b Additive effects of the marker calculated as ((average of 318) – (average of IR-44595))/2. Positive values mean that the IR-44595 allele decreased the trait value.

c Percentage of variance explained by the QTL.

d Non-significant (suggestive) QTL.

For Na^+^ sheath-blade ratio, no significant QTLs were detected. However, two suggestive QTL for Na^+^ sheath-blade ratio (*qNSBR4*, LOD = 3.81, *qNSBR11*, LOD = 3.59, i.e. slightly below the 5% threshold (LOD = 3.83) and above the 10% threshold (LOD = 3.48)) were detected on chromosome 4 and 11 (**Figure 2A**, **Table 1**). At the similar positions to *qNSBR11*, significant QTLs for Na^+^ concentration in leaf blades (*qBNC11*), Na^+^ concentration in leaf sheaths (*qSHNC11*), Na^+^ concentration in shoots (*qSNC11*) and K^+^-Na^+^ ratio in leaf blades (*qBKNR11*) were also detected (**Figures 2B–D**, **Table 1**). At these loci on chromosome 11, the IR-44595 alleles decreased Na^+^ concentration in leaf blades, leaf sheaths and shoots and enhanced Na^+^ sheath-blade ratio and K^+^-Na^+^ ratio in leaf blades (**Table 1**). On the other hand, at the locus of *qNSBR4* on chromosome 4, the 318 allele enhanced Na^+^ sheath-blade ratio (**Table 1**). A suggestive QTL for Na^+^ concentration in shoots (*qSNC4*) was also detected on chromosome 4 (**Figure 2D**, **Table 1**).

On chromosome 1, significant QTLs for Na^+^ concentration in shoots (*qSNC1.1*, *qSNC1.2*) were detected (**Figure 2D**, **Table 1**). At the similar locus with *qSNC1.1*, significant QTLs for Na^+^ concentration in leaf sheaths (*qSHNC1*) were also detected (**Figure 2C**, **Table 1**). At both loci, the 318 alleles decreased Na^+^ concentration in leaf sheaths and shoots (**Table 1**).

### Trait correlation in the F_2_ population

**Figure 3:**
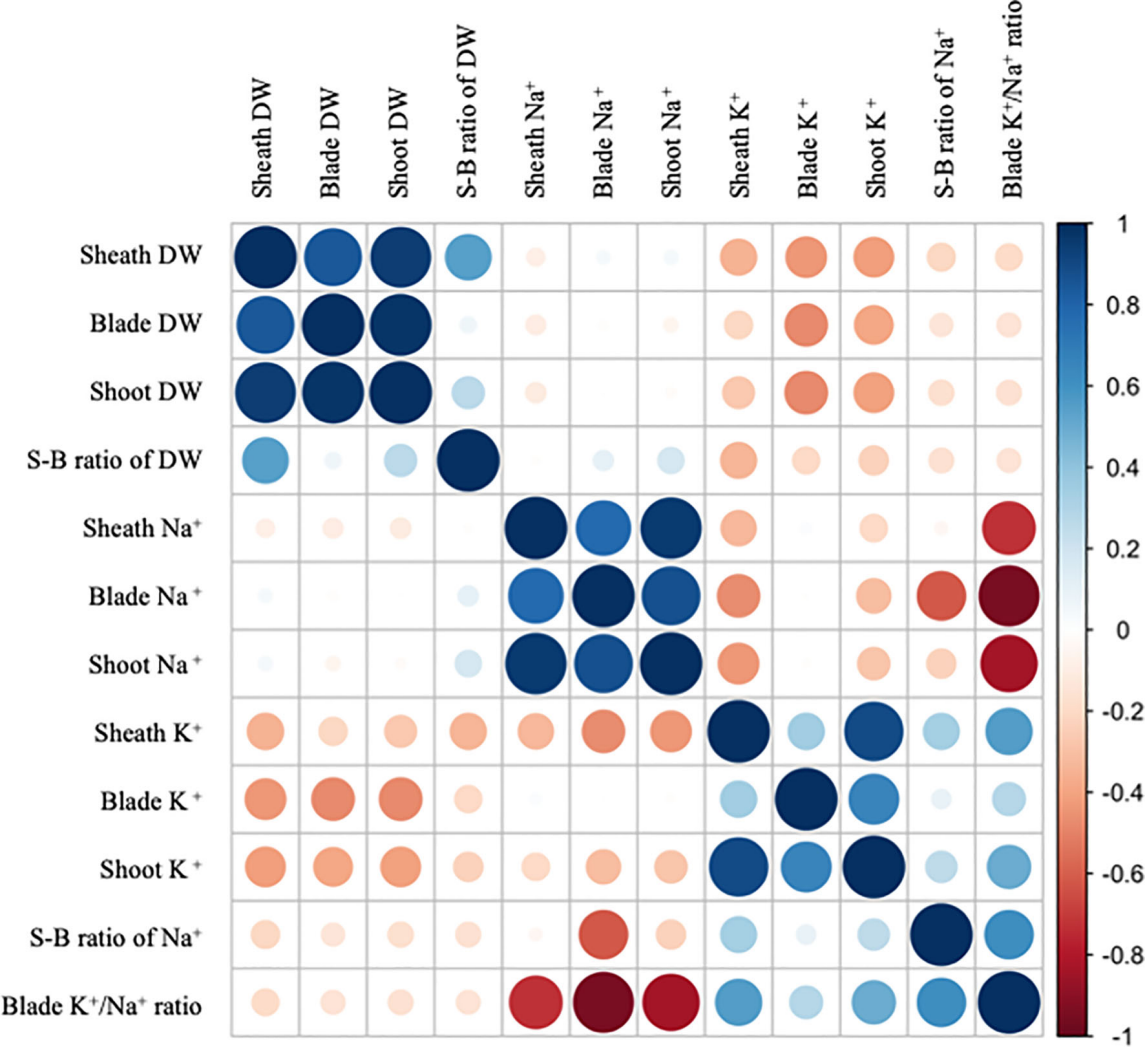
Correlations of phenotypes. Colors of dots indicate the Spearman correlation coefficients among traits in F_2_ families at seedling stage in the presence of 30 mM NaCl in hydroponics imposed for one week corresponding to the bar on the right. S-B ratio: Sheath-blade ratio.

Correlation analysis in the F_2_ population showed that Na^+^ sheath-blade ratio has a negative correlation with Na^+^ concentration in leaf blades but little correlation with Na^+^ concentration in leaf sheaths and shoots (**Figure 3**). Na^+^ concentration in leaf blades showed a positive correlation with Na^+^ concentration in shoots as well as leaf sheaths, and negative correlation with K^+^-Na^+^ ratio in leaf blades.

### QTL for biomass-related traits under salinity

In this study, some significant QTLs were detected for biomass-related traits under salinity on chromosome 1, 2, 5 and 12 (**Supplementary Table S1**). The sheath-blade ratio of dry weight is theoretically important for salt removal in leaf sheaths since larger leaf sheaths accumulate more amounts of salt, which might decrease salt entry into leaf blades. However, the sheath-blade ratio of dry weight has little correlation with either Na^+^ sheath-blade ratio or Na^+^ concentration in leaf blades in this study (**Figure 3**). This indicates that larger leaf sheaths did not relate to Na^+^ removal ability in leaf sheaths or Na^+^ accumulation level in leaf blades.

### Variation in Na^+^ removal ability in leaf sheaths within plants

**Figure 4:**
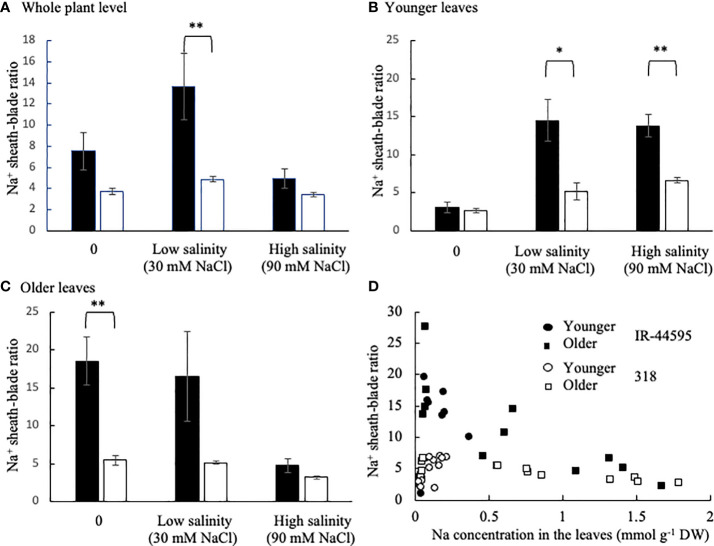
Na^+^ sheath-blade ratio at the whole plant level and in the younger and older leaves of IR-44595 and 318 plants. Plants were grown for 19 days in soil culture then treated with low (30 mM) and high (90 mM) concentration of NaCl for further 3 weeks. **(A)** Na^+^ sheath-blade ratio at the whole plant level, **(B)** in younger leaves, **(C)** in older leaves, are shown. The relationship between Na^+^ concentration in the leaves including leaf blades and leaf sheaths and the Na^+^ sheath-blade ratio is also shown **(D)**. Data are mean ± the standard error (n = 4). * and ** indicate significant difference at P<0.05 and 0.1 between IR-44595 and 318 under each treatment (Student’s *t*-test).

To determine whether the varietal difference in Na^+^ removal ability in leaf sheaths is significant under low salinity but not under high salinity, the Na^+^ sheath-blade ratio was determined at the whole plant level and in two parts (younger and older leaves) separately in IR-44595 and 318 under low (30 mM) and high (90 mM) NaCl treatment (**Supplementary Figure 1**). The Na^+^ sheath-blade ratio at the whole plant level was significantly higher in IR-44595 compared with 318 under low salinity (**Figure 4A**). However, the Na^+^ sheath-blade ratio at the whole plant level was comparable between two varieties under high salinity. On the other hand, when separating younger and older leaves to estimate the Na^+^ sheath-blade ratio, the younger parts of IR-44595 showed higher Na^+^ sheath-blade ratios compared with 318 under both low and high salinity (**Figure 4B**). The Na^+^ sheath-blade ratio in older leaves was not significantly different between IR-44595 and 318 under both low and high salinity (**Figure 4C**).

The relationship between Na^+^ concentration in the leaves including leaf blades and leaf sheaths and the Na^+^ sheath-blade ratio (**Figure 4D**) indicated that IR-44595 showed high Na^+^ sheath-blade ratio when Na^+^ concentration in the leaves was low regardless of the leaf age. However, the Na^+^ sheath-blade ratio decreased with increasing Na^+^ concentration in the leaves. On the other hand, 318 showed a constantly low Na^+^ sheath-blade ratio compared with IR-44595 regardless of the Na^+^ concentration in the whole leaves.

## Discussion

In this study, two suggestive QTLs for Na^+^ sheath-blade ratio (*qNSBR4* and *qNSBR11*) were detected. Because Na^+^ removal ability in leaf sheath is involved in decreasing Na^+^ accumulation in leaf blades in the F_2_ population (**Figure 3**), it is postulated that these QTLs may contribute to salt tolerance by decreasing Na^+^ entry into leaf blades. At the *qNSBR11* locus on chromosome 11, QTLs for Na^+^ concentrations in shoots (*qSNC11*), leaf blades (*qBNC11*) and leaf sheaths (*qSHNC11*) were also detected (**Table 1**). Since the QTL for Na^+^ concentration in shoots may be attributed to restriction of Na^+^ entry from roots to shoots, the *qNSBR11* locus may regulate Na^+^ removal ability not only in leaf sheaths but also in roots. There is no reported QTL related to Na^+^ homeostasis in this locus (Singh et al., 2021). Therefore, *qNSBR11* may be a good candidate to be used for molecular breeding of salt tolerant rice by improving salt removal ability in leaf sheaths and possibly roots as well.

On the other hand, on chromosome 4, 318 allele of *qNSBR4* increases Na^+^ sheath-blade ratio. This QTL was close to the locus of *qSNC4*, a suggestive QTL for Na^+^ concentration in shoots, although these are not overlapped (**Table 1**). The *qNSBR4* overlapped with *qNaK4.1* that was found from the F_2_ population between IR 64 and IR 4630-22-2-5-1-3 as a QTL for Na^+^/K^+^ ratio (Singh et al., 2021). *OsClo5* has been reported as a salt tolerance-related gene at the 25.4 Mb region on chromosome 4 (Negrão et al., 2011) and is close to the *qNSBR4* locus (24.1 to 24.8 Mb on chromosome 4). OsClo5 protein binds calcium and phospholipids *in vitro* and functions as a transcriptional co-repressor by interacting with OsDi19-5 to negatively affect salt stress tolerance in rice seedlings (Jing et al., 2021). There is no evidence of the involvement of *OsClo5* in the regulation of Na^+^ homeostasis under salinity.

Major QTLs for Na^+^ concentration in shoots and leaf sheaths were detected on chromosome 1 (**Figure 2**). The regions of *qSNC1.1*, *qSNC1.2*, *qSHNC1* on chromosome 1 has been reported as a meta-QTL for salt tolerance (Singh et al., 2021) and QTLs regulating Na^+^, K^+^ and Cl^-^ concentrations in shoots and roots and the Na^+^/K^+^ ratio such as *qSKC1* and *qSaltol* (Lin et al., 2004; Ren et al., 2005; Thomson et al., 2010) are overlapped with those major QTLs in this study. Salt tolerance-related genes such as *OsHKT8/OsHKT1;5*, *SalT* and *OsCIPK8* are localized in this region (Negrão et al., 2011). CIPK (Calcineurin B-like protein interaction protein kinase) is involved in transducing calcium signals by phosphorylating downstream signaling components (Liu et al., 2000). Although it is not known yet whether OsCIPK8 functions in Na^+^ homeostasis regulation, it may have some physiological roles under salinity since the transcript level is increased by salt treatment (Xiang et al., 2007). OsHKT1;5 mediates the Na^+^ unloading from xylem vessels into the surrounding xylem parenchyma cells in roots (Ren et al., 2005) and leaf sheaths (Kobayashi et al., 2017). It was speculated that *qSNC1.1*, *qSNC1.2* and *qSHNC1* found in this study might be attributed to these genes especially *OsHKT1;5*. It was interesting that no QTL for Na^+^ sheath-blade ratio was detected in the region on chromosome 1. Although *OsHKT1;5* gene has been reported to be involved in Na unloading from xylem vessels in leaf sheaths (Kobayashi et al., 2017), this gene might not be related to the genotypic variation of Na^+^ removal ability in leaf sheaths between IR-44595 and 318.

It was found that the Na^+^ sheath-blade ratio was variable within plants and was higher in the younger leaves with lower Na^+^ accumulated in IR-44595 even under high salinity (**Figure 4**). Since Na^+^ is sequestrated more in older leaves than in younger ones (Mitsuya et al., 2002; Neang et al., 2019), Na^+^ in xylem vessels may be low enough in younger leaves even under high salinity so Na^+^ was efficiently removed in leaf sheaths in the younger leaves of IR-44595 under high salinity. Therefore, the Na^+^ removal ability in leaf sheaths is important to protect young leaf blades where photosynthesis is active, in coordination with Na^+^ sequestration ability in older leaves. This study also suggests that, under high salinity, it is important to evaluate Na^+^ removal ability in leaf sheaths in the younger leaves, as Na^+^ removal ability in leaf sheaths at the whole plant level under high salinity may underestimate the ability and misread varietal differences of the ability.

In summary, the QTLs for Na^+^ removal ability in leaf sheaths detected in this study may help to develop salt tolerant rice varieties through marker-assisted breeding. Also, using the QTL information in this study, more detailed study is necessary to identify specific genes regulating Na^+^ removal ability in leaf sheaths in each locus. Also, to see the effect of single QTL in uniform genetic backgrounds, producing near isogenic lines (NILs) and residual heterozygous lines (RHLs) is on-going.

## Data availability statement

The datasets presented in this study can be found in online repositories. The names of the repository/repositories and accession number(s) can be found below: https://www.ddbj.nig.ac.jp/, DRA014510.

## Author contributions

SN and SM designed the experiments. IG, RK, VR, KD and SM performed most of experiments and analyzed the data. NS, KD, MT and AY assisted in experiments and discussed the results. SM wrote the manuscript and all authors revised the manuscript. All authors read and approved the final manuscript.

## Funding

This work was supported in part by JSPS KAKENHI Grant Numbers 19H02942 and 22H02325 to SM and Science and Technology Research Partnership for Sustainable Development (SATREPS) “Rice genome breeding system for developing rice plant for non-irrigated area” to KD. This work was partially supported by Nagoya University Research Fund.

## Acknowledgments

We thank Genebank at IRRI for providing us the seeds of the rice varieties we used in this study.

## Conflict of interest

The authors declare that the research was conducted in the absence of any commercial or financial relationships that could be construed as a potential conflict of interest.

## Publisher’s note

All claims expressed in this article are solely those of the authors and do not necessarily represent those of their affiliated organizations, or those of the publisher, the editors and the reviewers. Any product that may be evaluated in this article, or claim that may be made by its manufacturer, is not guaranteed or endorsed by the publisher.
