# Supplementary material for: QTL analysis for sodium removal ability in rice leaf sheaths under salinity using an IR-44595/318 F2 population
Source: Front Plant Sci. 2022 Oct 11;13:1002605. doi: 10.3389/fpls.2022.1002605 (PMC9592983; doi:10.3389/fpls.2022.1002605)
Supplement: Supplementary file 1 [file DataSheet_1.pdf]

**Supplementary Table 1.** QTL detected in $F_2$ generation for traits for morphological traits from the cross of IR-44595 and 318 in the presence of 30 mM NaCl in hydroponics at the seedling stage based on interval mapping

| QTL | Chr[a] | Trait | Marker | Position (Mb) | LOD | AE[b] | PVE[c] (%) |
|---|---|---|---|---|---|---|---|
| *qSDW1* | 1 | Shoot dry weight | S01_37831300 | 27.8 | 4.96 | 17.59 | 12.71 |
| *qSHDW1* | 1 | Leaf sheath dry weight | S01_37831300 | 37.8 | 8.03 | 9.23 | 19.76 |
| *qDWSBR1* | 1 | Sheath-blade ratio of dry weight | S01_37831300 | 37.8 | 6.16 | 0.048 | 15.54 |
| *qSDW2* | 2 | Shoot dry weight | S02_26511771 | 26.5 | 5.19 | 16.48 | 13.26 |
| *qBDW2* | 2 | Leaf blade dry weight | S02_26501890 | 26.5 | 5.75 | 10.92 | 14.58 |
| *qSDW5* | 5 | Shoot dry weight | S05_18531122 | 18.5 | 6.73 | -20.74 | 16.85 |
| *qBDW5* | 5 | Leaf blade dry weight | S05_18531122 | 18.5 | 5.74 | -12.07 | 14.56 |
| *qSHDW5* | 5 | Leaf sheath dry weight | S05_18531122 | 18.5 | 6.87 | -8.66 | 17.16 |
| *qDWSBR12* | 12 | Sheath-blade ratio of dry weight | S12_26666562 | 26.7 | 4.04 | -0.038 | 10.48 |

[a]Chromosome

[b]Additive effects of the marker calculated as ((average of 318) – (average of IR-44595))/2. Positive values mean that the IR-44595 allele decreased the trait value..

[c]Percentage of variance explained by the QTL

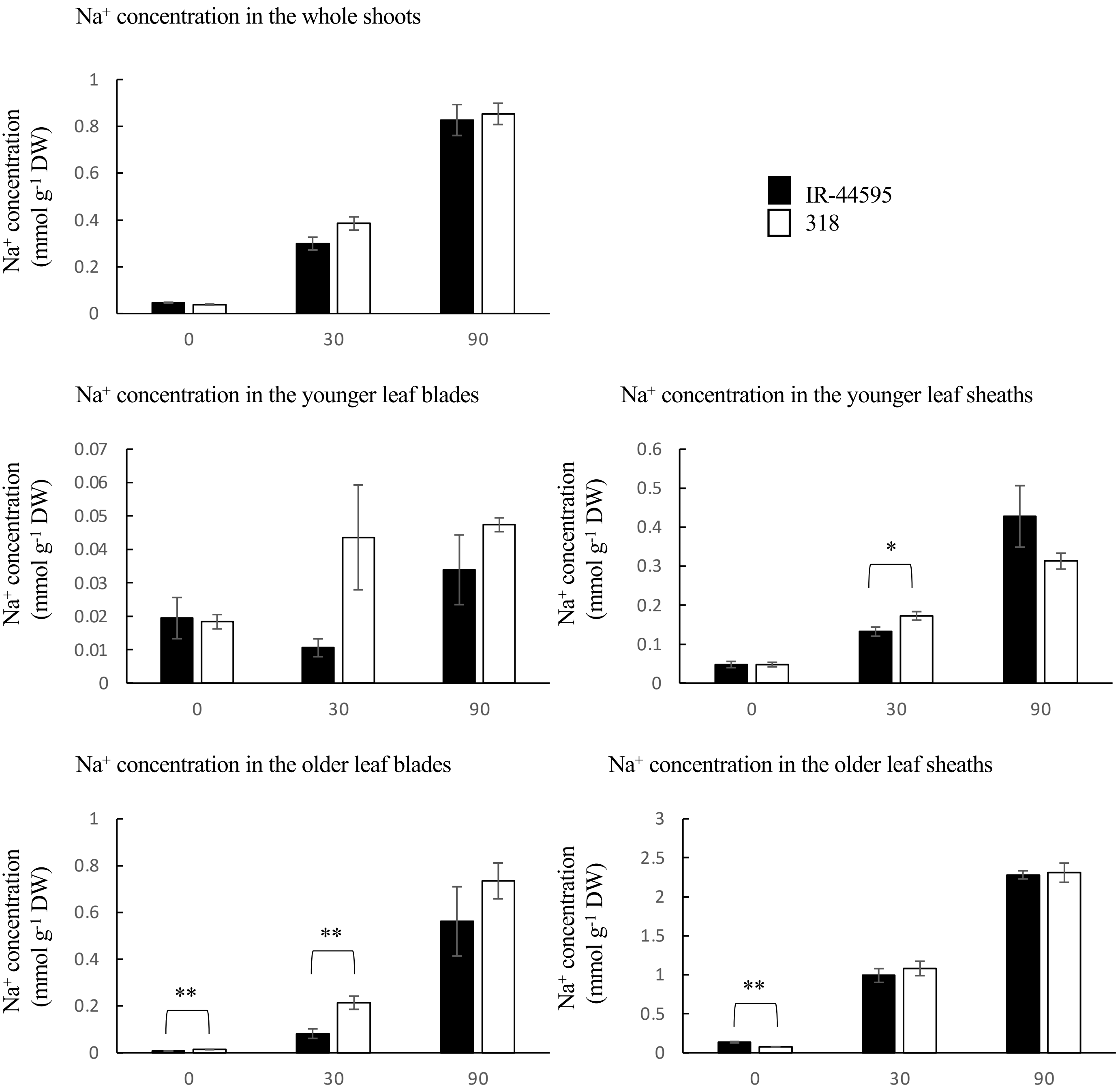


**Supplementary Figure 1.** $Na^+$ concentration in the shoots of IR-44595 and 318 plants grown in soil culture. Plants were grown for 19 days then treated with low (30 mM) and high (90 mM) NaCl concentration for further 3 weeks. Non-etiolated leaves were harvested and divide into younger and older parts. Data are mean of three replications ± the standard error. * and ** indicate significant difference at P<0.05 and 0.1 between IR-44595 and 318 under each treatment (Student's *t*-test).

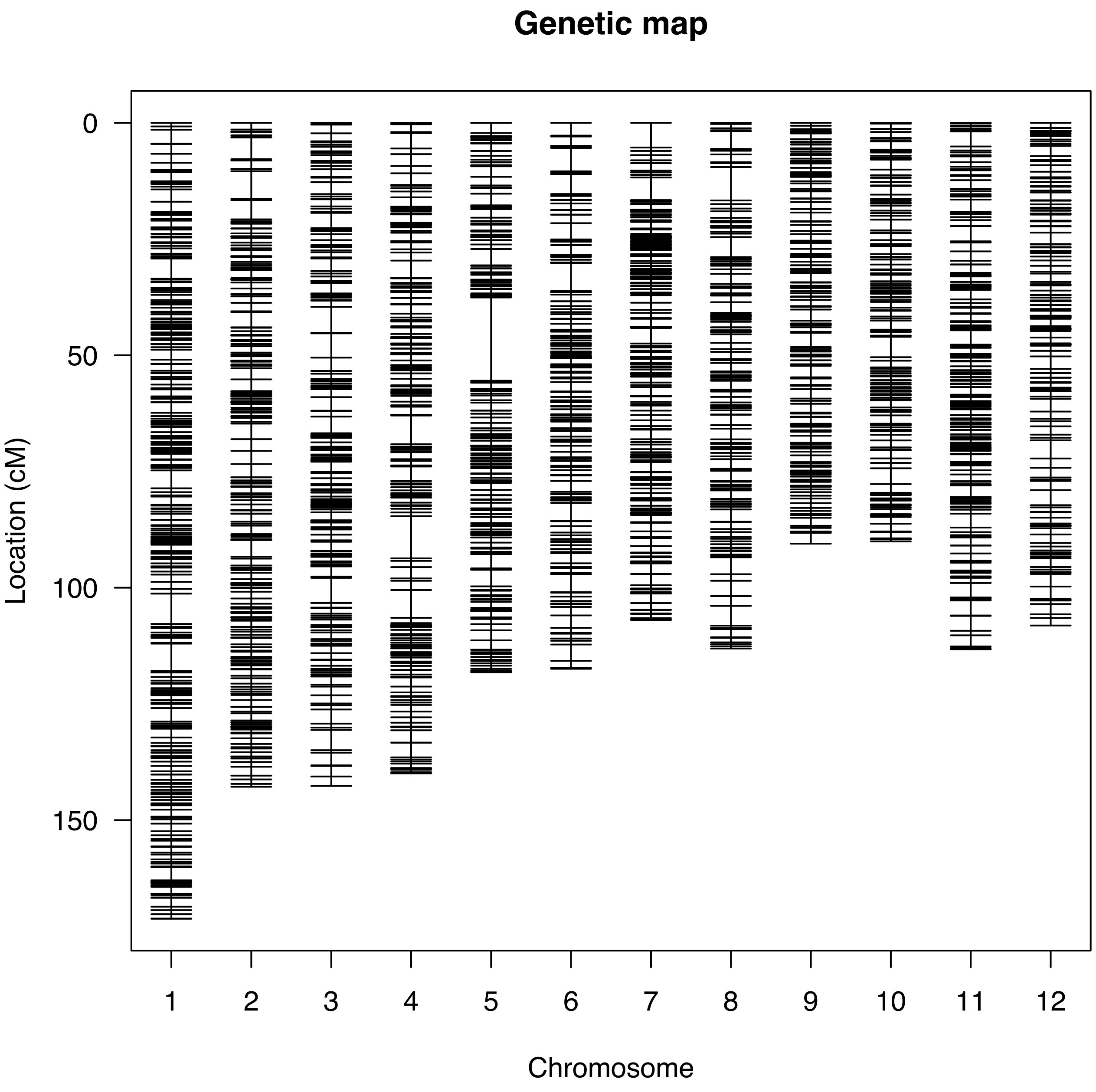


**Supplemental Figure 2.** A linkage map showing the locations of single nucleotide polymorphism markers (SNPs) in the population used in this study.

**Supplemental Figure 3.**
Results of QTL analysis. Results of a trait are shown in each page.

Top panels show the results of interval mapping for the trait. Dotted and solid lines indicate the threshold at 5 and 10% levels, respectively.

Middle panels show a scan of additive effects corresponding tot eh top panel. The values were calculated as follows: ((Average of 318) – (average of IR-44595))/2.

Bottom panels show dot plots of trait values in each variety at the marker shown on the top of each panel. For example, a marker name "S04_24088589" means a single nucleotide polymorphism marker at 24088689 bp position on chromosome 4 based on IRGSP1 rice reference sequence. AA: IR-44595 and BB: 318 varieties. Red dots are imputed trait values. Significant and suggestive QTLs are shown.

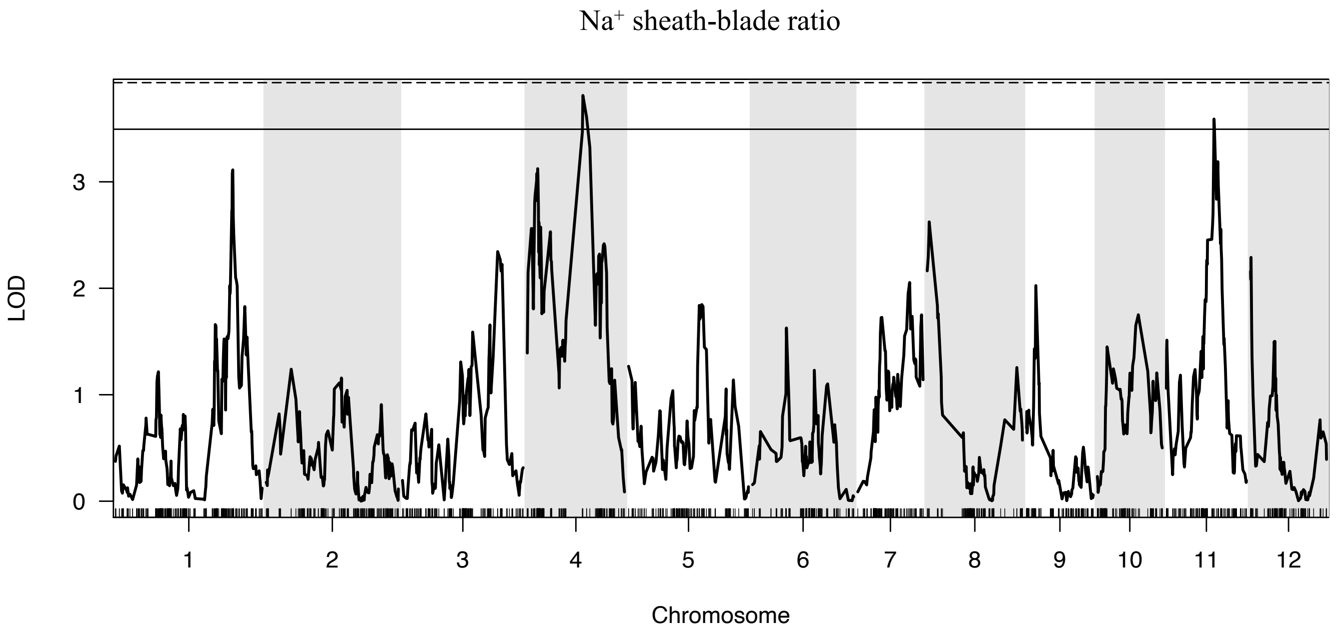

Na+ sheath-blade ratio
LOD
0
1
2
3
1 2 3 4 5 6 7 8 9 10 11 12
Chromosome


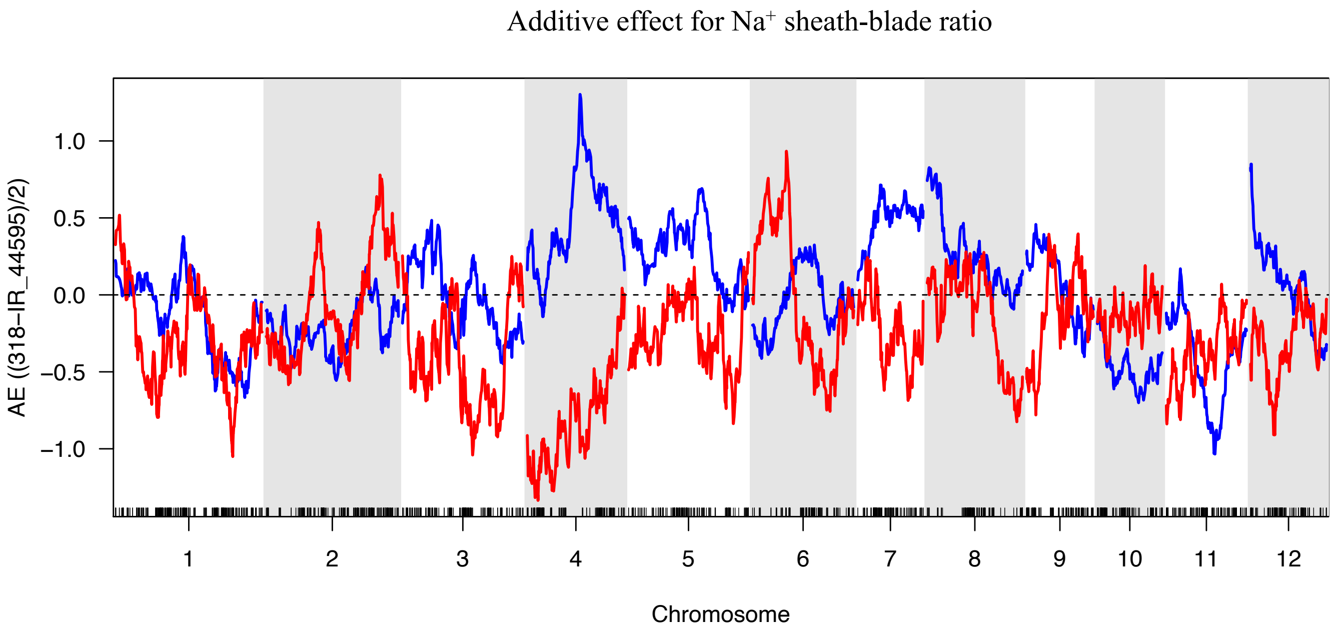

Additive effect for Na+ sheath-blade ratio
AE ((318−IR_44595)/2)
−1.0
−0.5
0.0
0.5
1.0
1 2 3 4 5 6 7 8 9 10 11 12
Chromosome


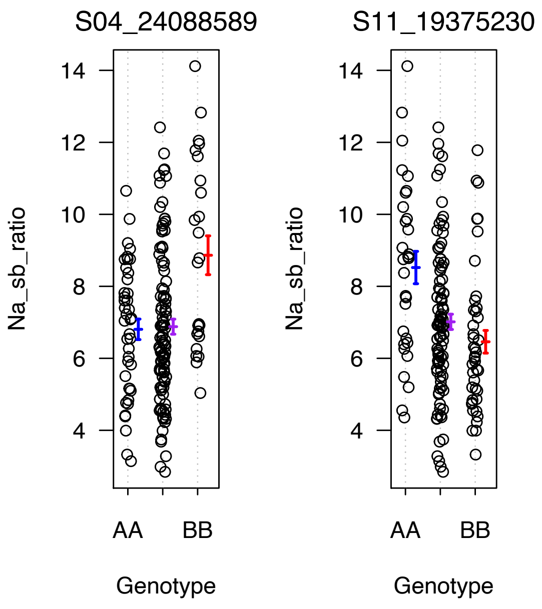

S04_24088589
S11_19375230
Na_sb_ratio
4
6
8
10
12
14
AA
BB
Genotype

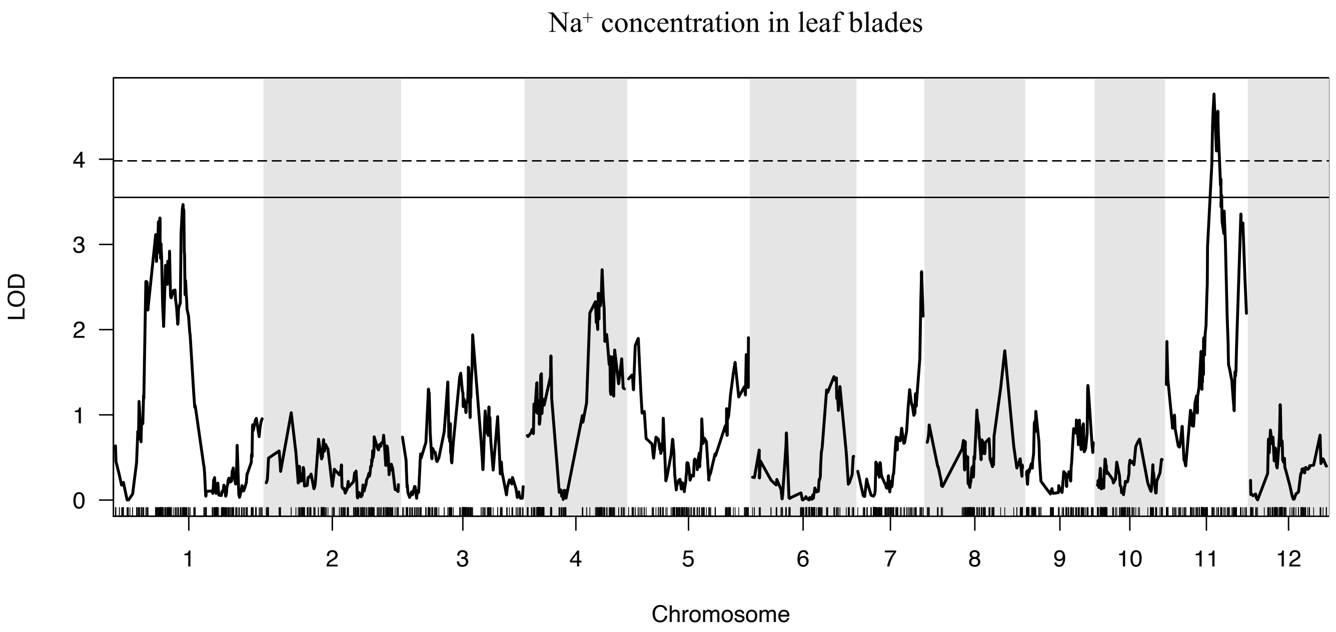
Na⁺ concentration in leaf blades
LOD
Chromosome
0
1
2
3
4
1
2
3
4
5
6
7
8
9
10
11
12

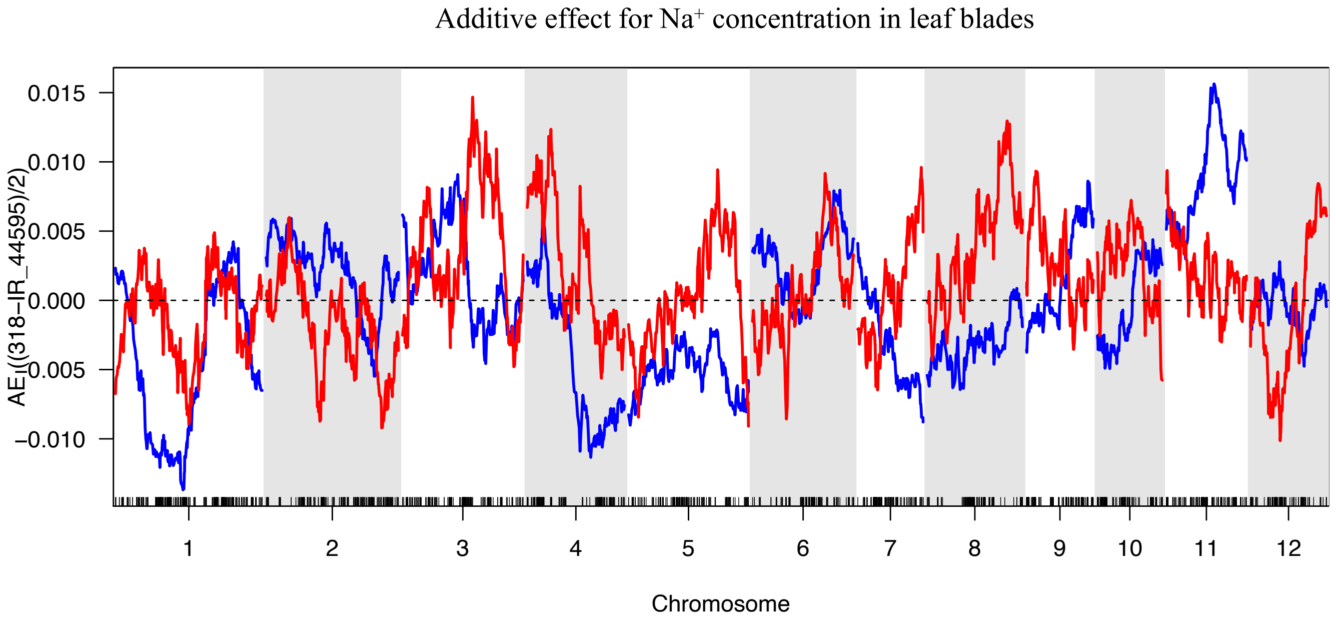
Additive effect for Na⁺ concentration in leaf blades
AE_((318−IR_44595)/2)
0.015
0.010
0.005
0.000
−0.005
−0.010
Chromosome
1
2
3
4
5
6
7
8
9
10
11
12

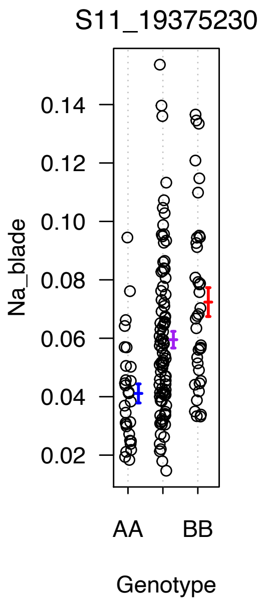
S11_19375230
Na_blade
0.14
0.12
0.10
0.08
0.06
0.04
0.02
AA
BB
Genotype

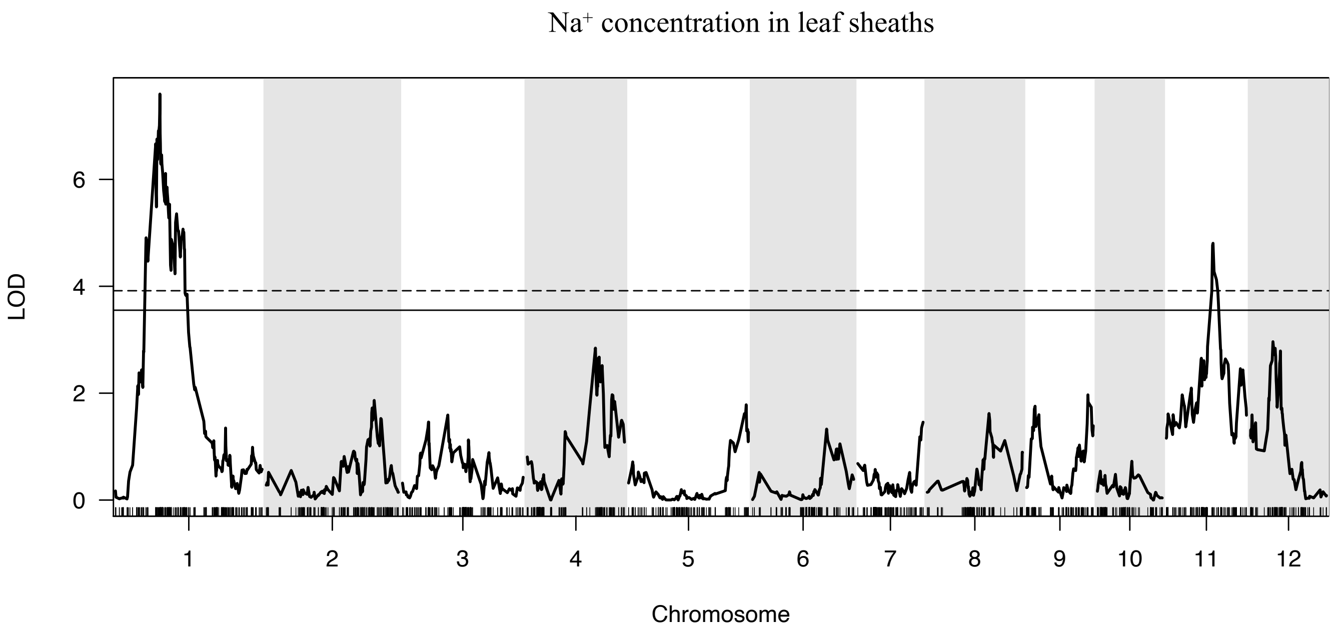
Na+ concentration in leaf sheaths
LOD
0
2
4
6
1
2
3
4
5
6
7
8
9
10
11
12
Chromosome

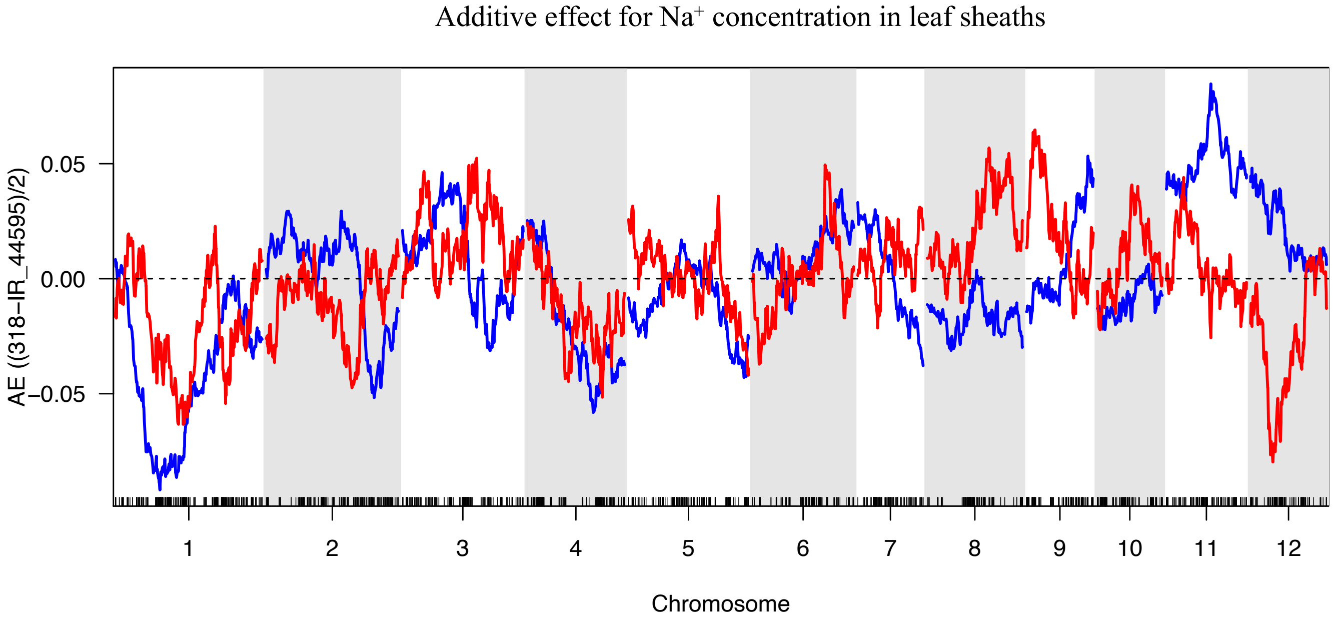
Additive effect for Na+ concentration in leaf sheaths
AE ((318−IR_44595)/2)
−0.05
0.00
0.05
1
2
3
4
5
6
7
8
9
10
11
12
Chromosome

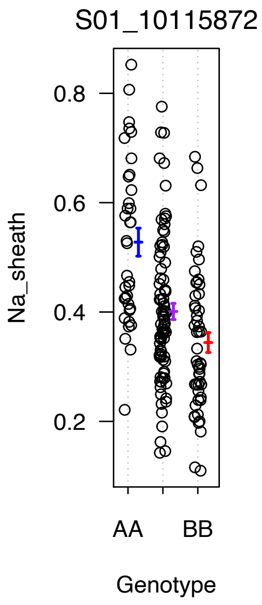
S01_10115872
Na_sheath
0.2
0.4
0.6
0.8
AA
BB
Genotype

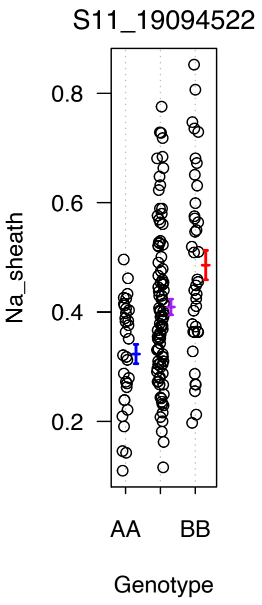
S11_19094522
Na_sheath
0.2
0.4
0.6
0.8
AA
BB
Genotype

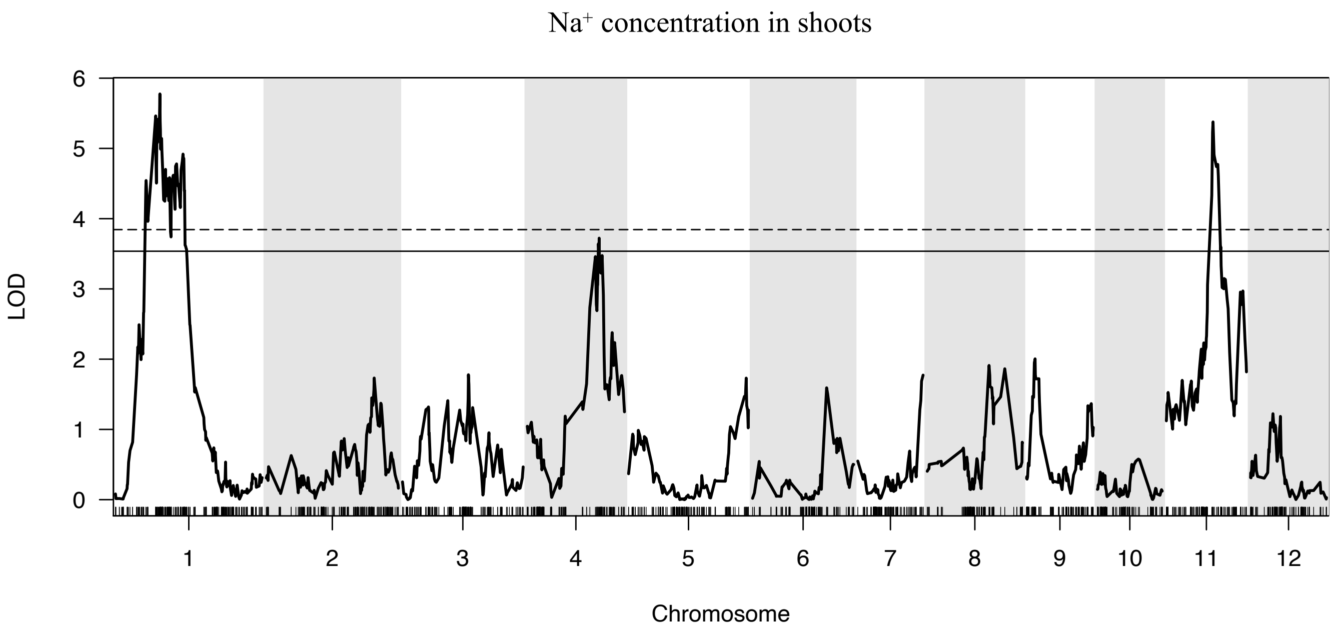
Na+ concentration in shoots
LOD
Chromosome
1 2 3 4 5 6 7 8 9 10 11 12

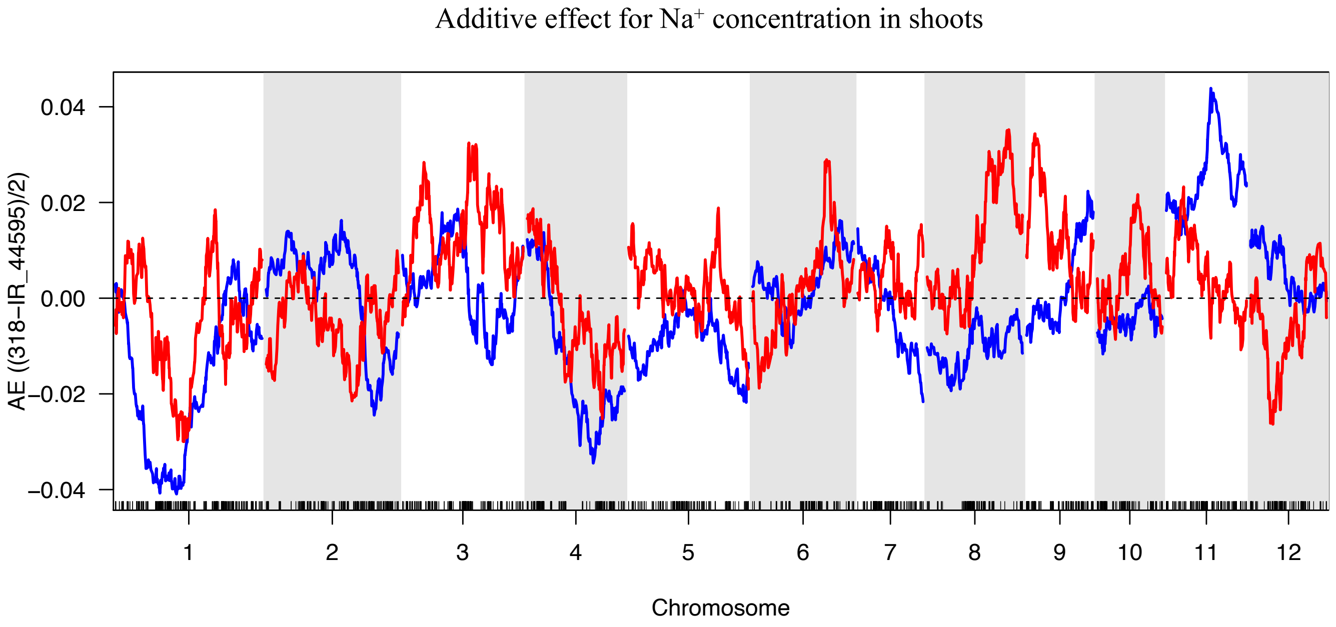
Additive effect for Na+ concentration in shoots
AE ((318−IR_44595)/2)
Chromosome
1 2 3 4 5 6 7 8 9 10 11 12

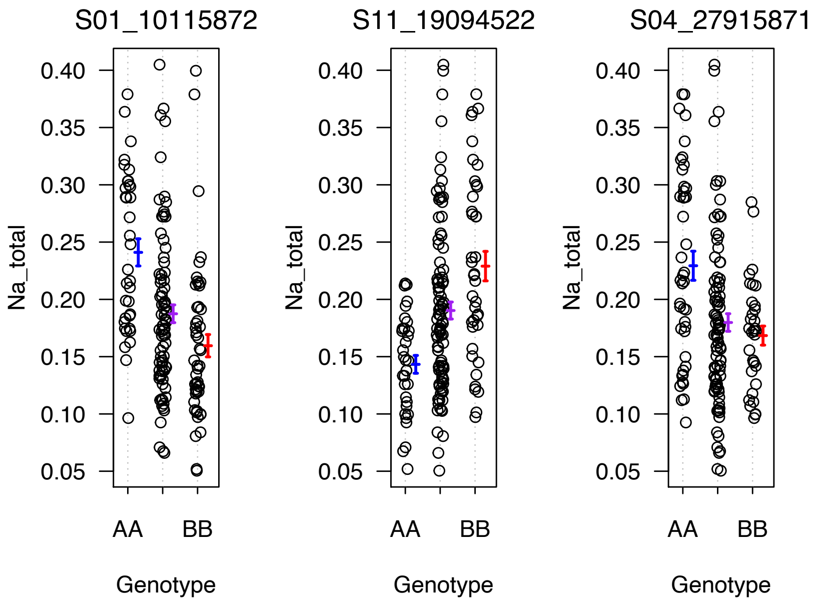
S01_10115872
S11_19094522
S04_27915871
Na_total
AA
BB
Genotype

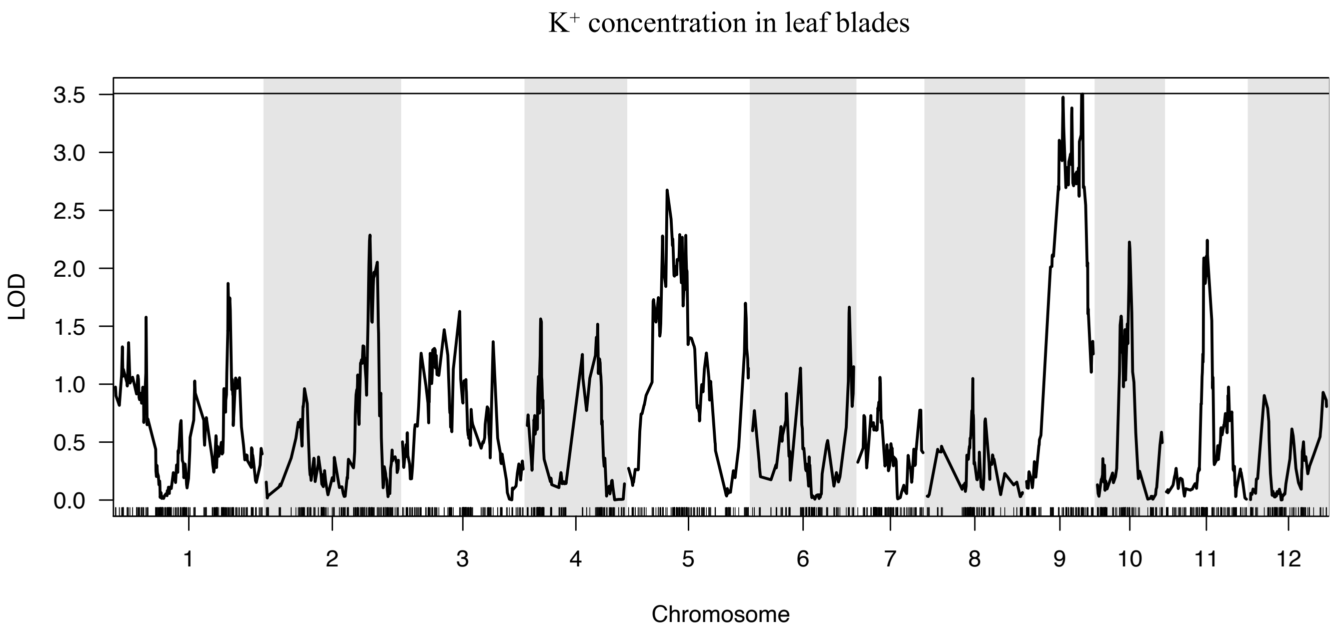
K+ concentration in leaf blades
LOD
3.5
3.0
2.5
2.0
1.5
1.0
0.5
0.0
1
2
3
4
5
6
7
8
9
10
11
12
Chromosome

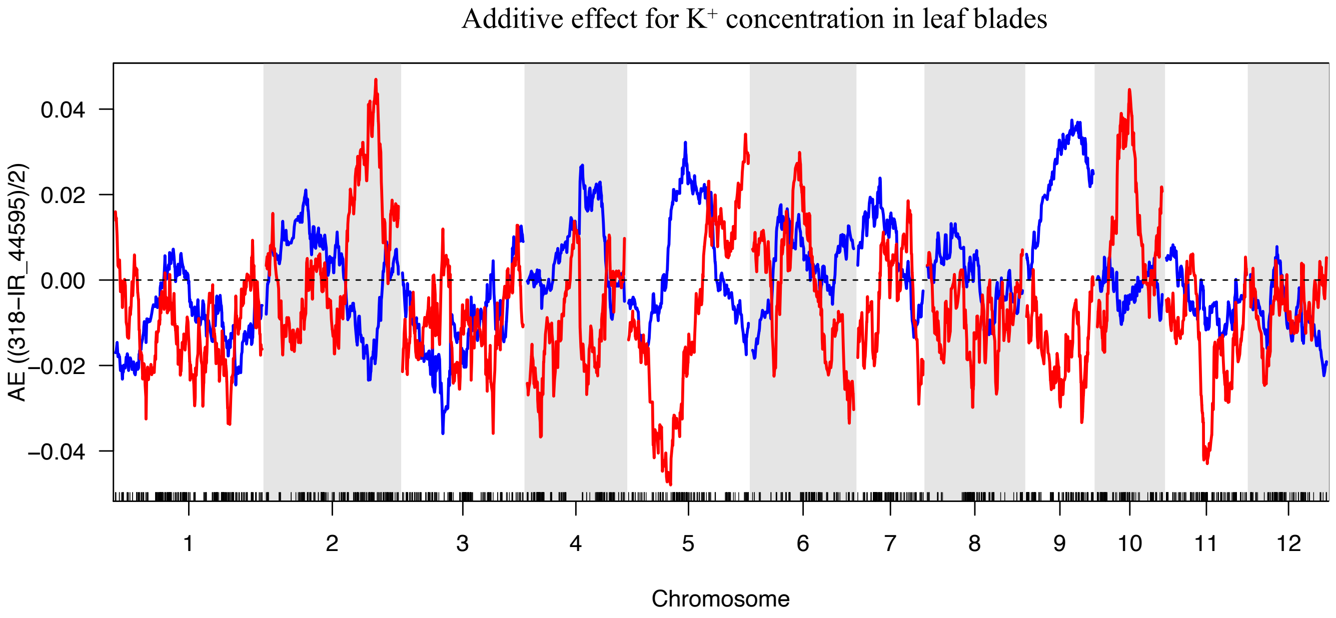
Additive effect for K+ concentration in leaf blades
AE_((318−IR_44595)/2)
0.04
0.02
0.00
−0.02
−0.04
1
2
3
4
5
6
7
8
9
10
11
12
Chromosome

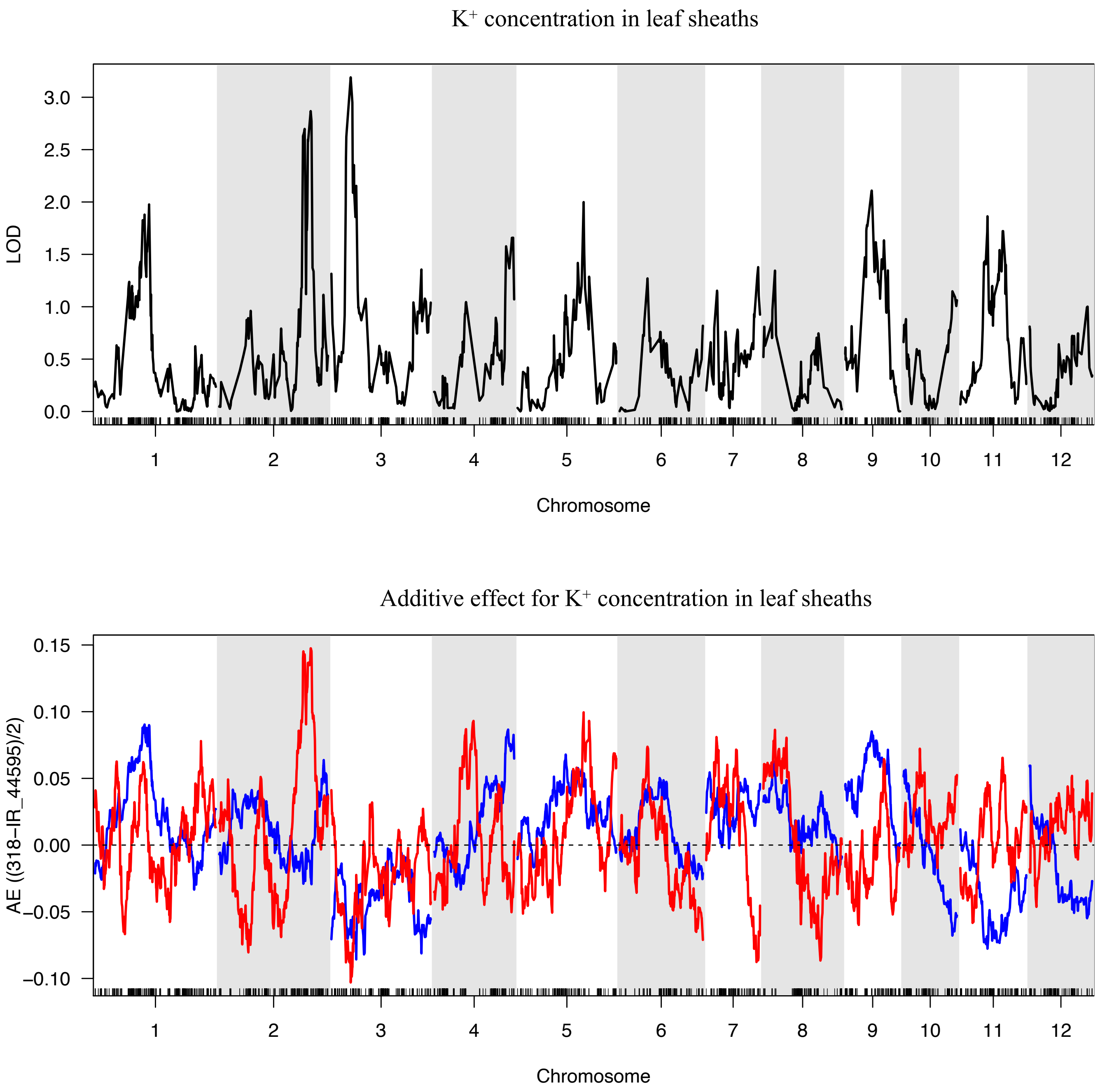

K+ concentration in leaf sheaths
LOD
3.0
2.5
2.0
1.5
1.0
0.5
0.0
1
2
3
4
5
6
7
8
9
10
11
12
Chromosome
Additive effect for K+ concentration in leaf sheaths
AE ((318−IR_44595)/2)
0.15
0.10
0.05
0.00
−0.05
−0.10
1
2
3
4
5
6
7
8
9
10
11
12
Chromosome

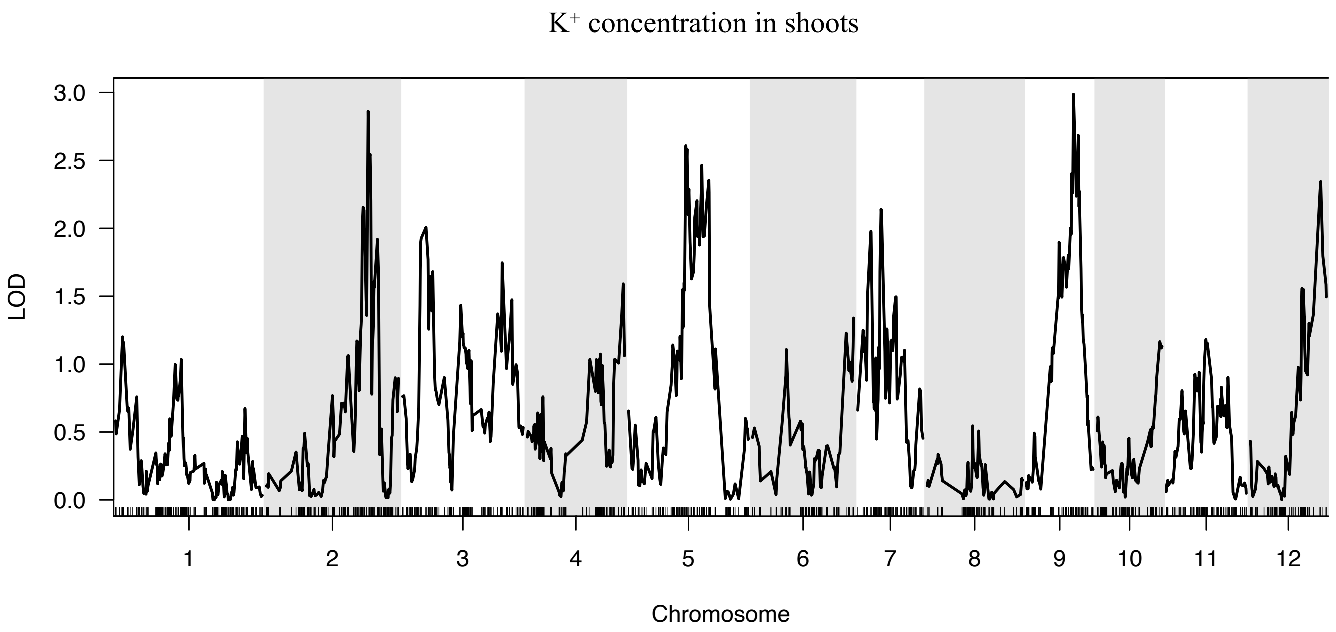
K+ concentration in shoots
LOD
3.0
2.5
2.0
1.5
1.0
0.5
0.0
1 2 3 4 5 6 7 8 9 10 11 12
Chromosome

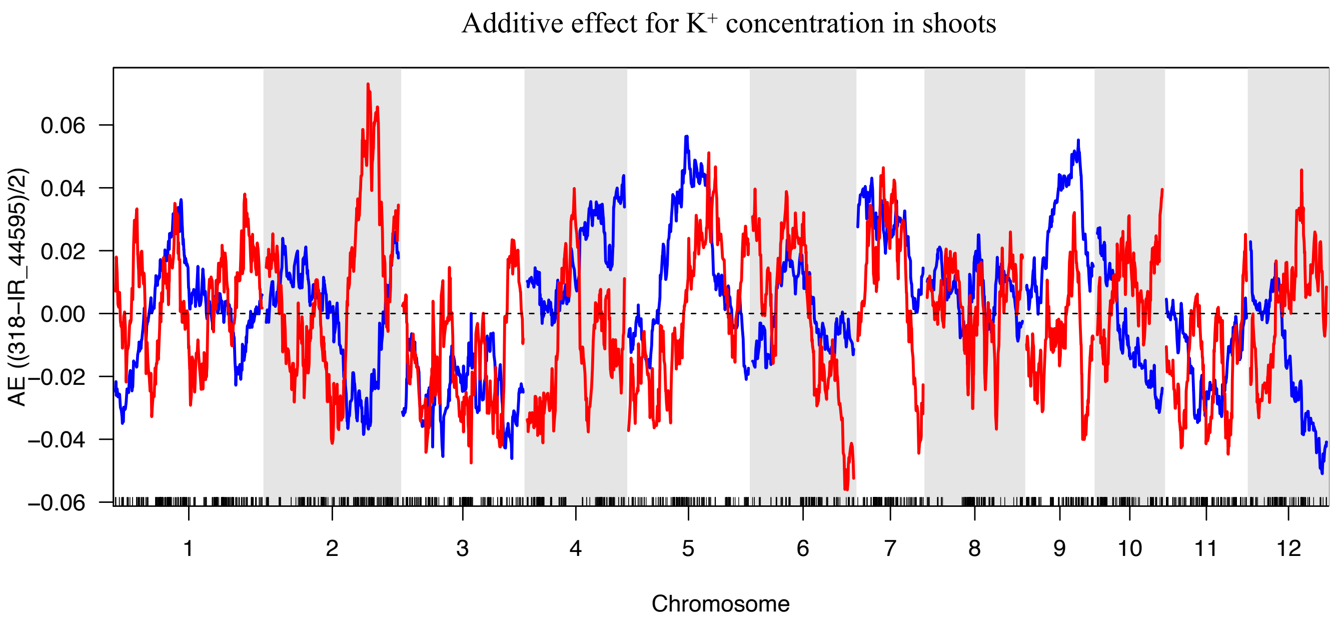
Additive effect for K+ concentration in shoots
AE ((318−IR_44595)/2)
0.06
0.04
0.02
0.00
−0.02
−0.04
−0.06
1 2 3 4 5 6 7 8 9 10 11 12
Chromosome

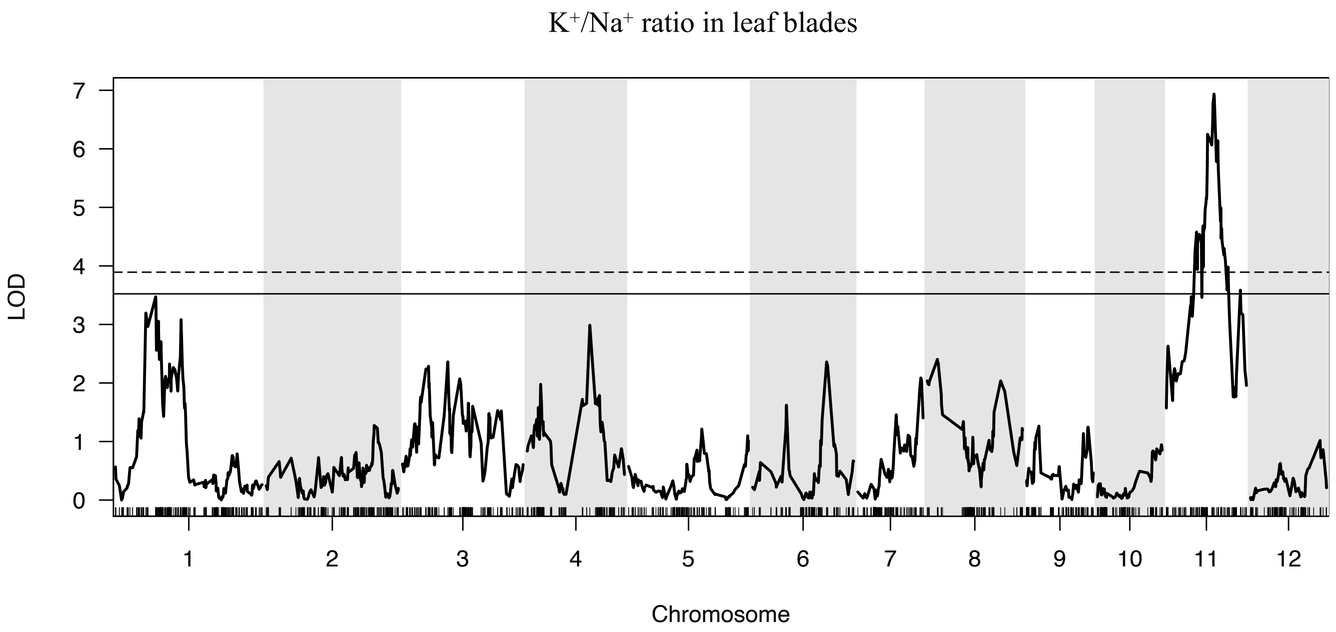
K+/Na+ ratio in leaf blades
LOD
Chromosome

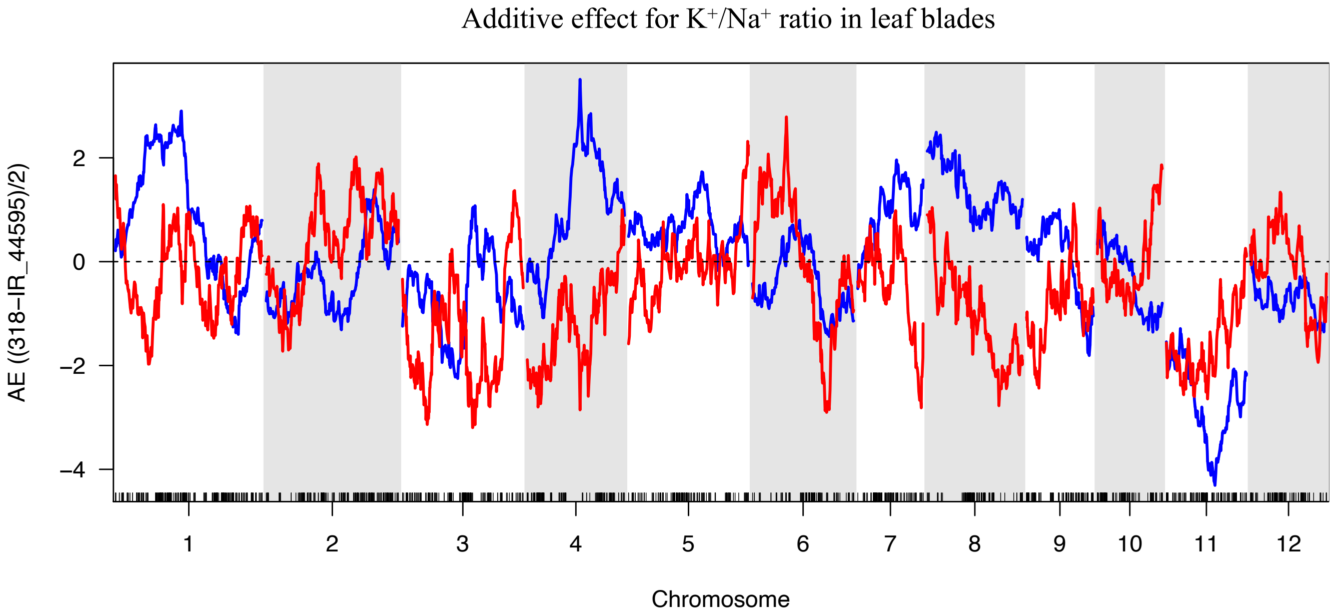
Additive effect for K+/Na+ ratio in leaf blades
AE ((318−IR_44595)/2)
Chromosome

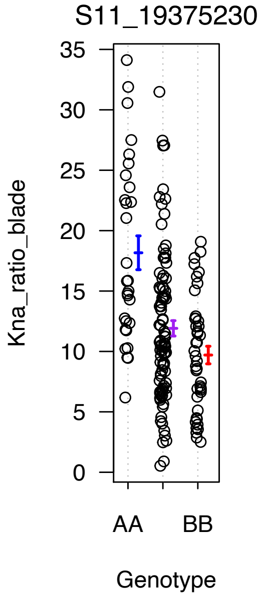
S11_19375230
Kna_ratio_blade
AA
BB
Genotype

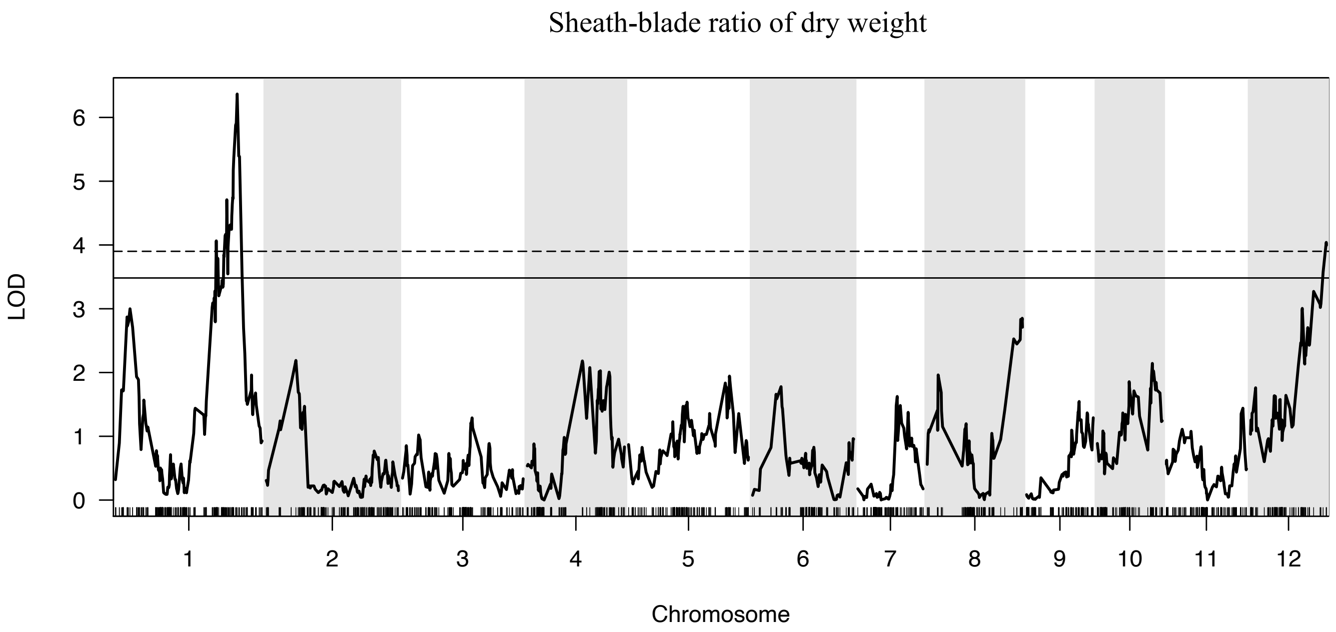
Sheath-blade ratio of dry weight
LOD
Chromosome

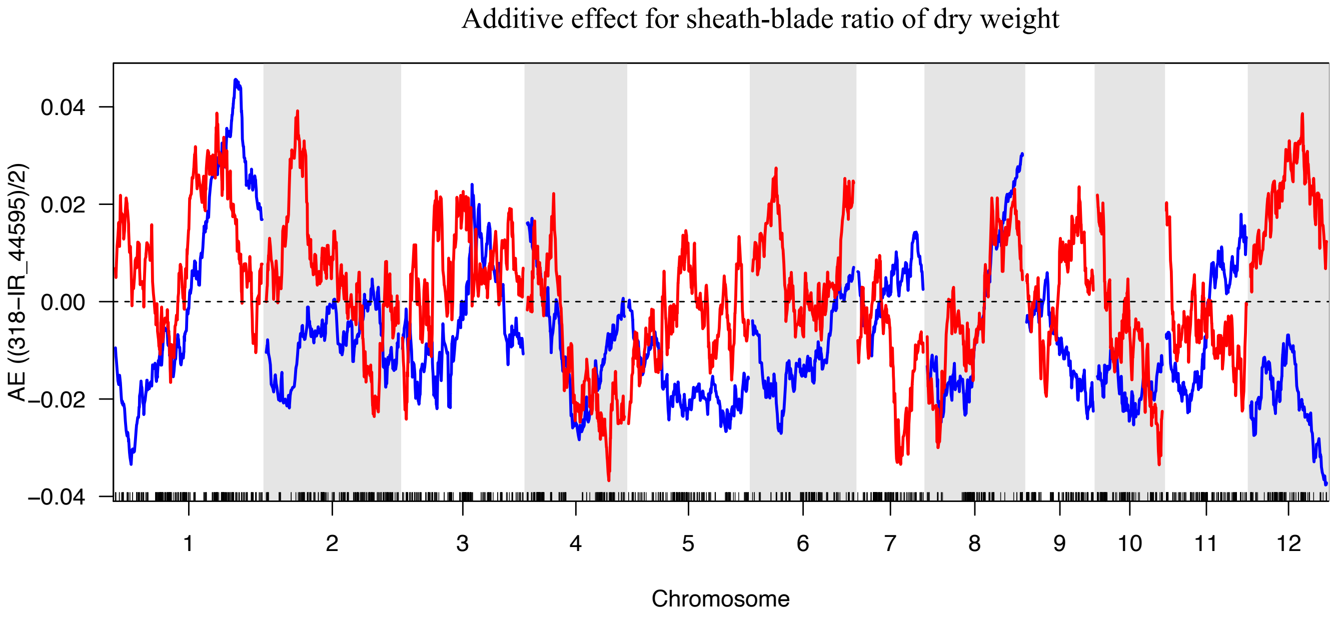
Additive effect for sheath-blade ratio of dry weight
AE ((318−IR_44595)/2)
Chromosome

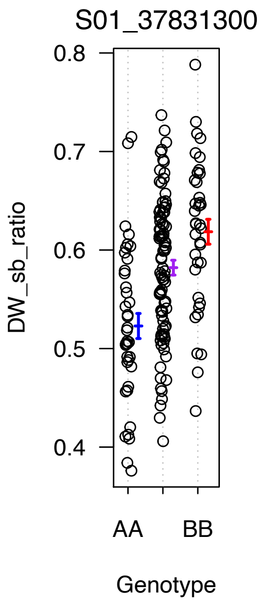
S01_37831300
DW_sb_ratio
AA
BB
Genotype

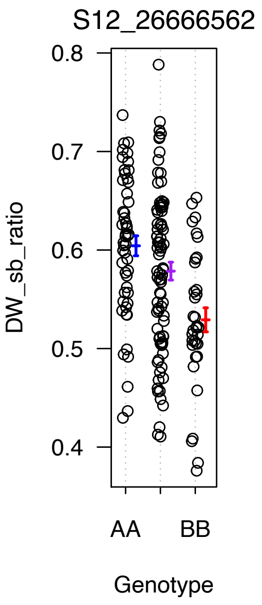
S12_26666562
DW_sb_ratio
AA
BB
Genotype

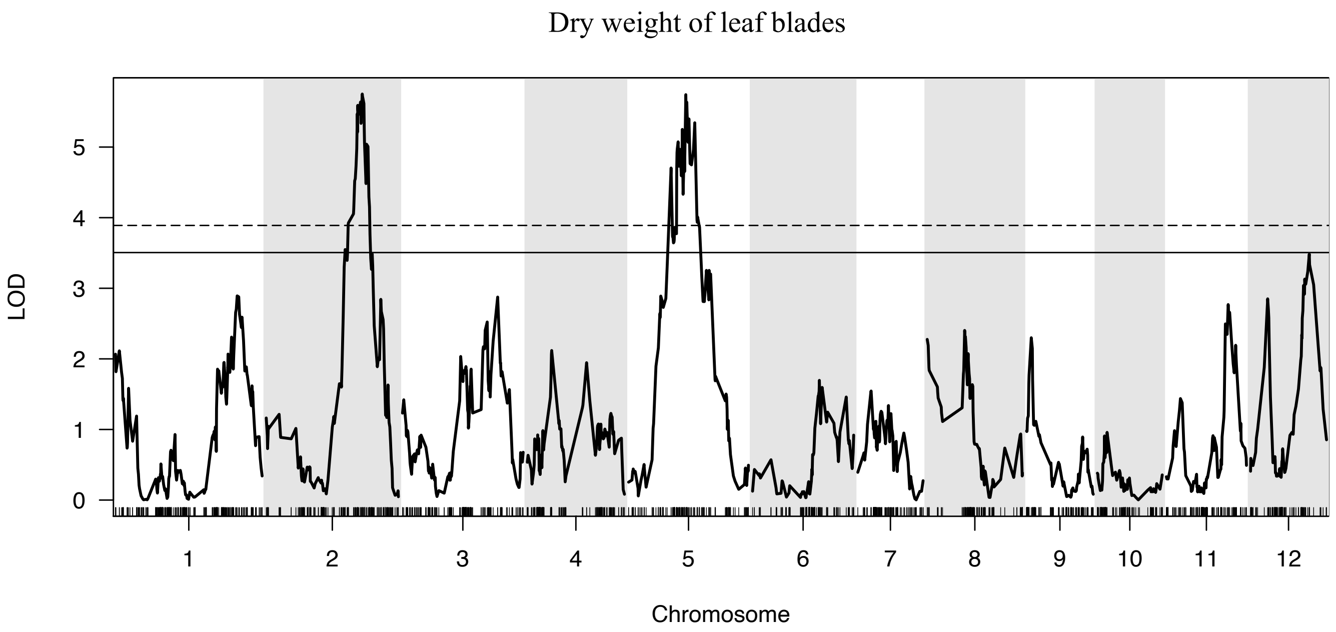
Dry weight of leaf blades
LOD
0
1
2
3
4
5
1
2
3
4
5
6
7
8
9
10
11
12
Chromosome

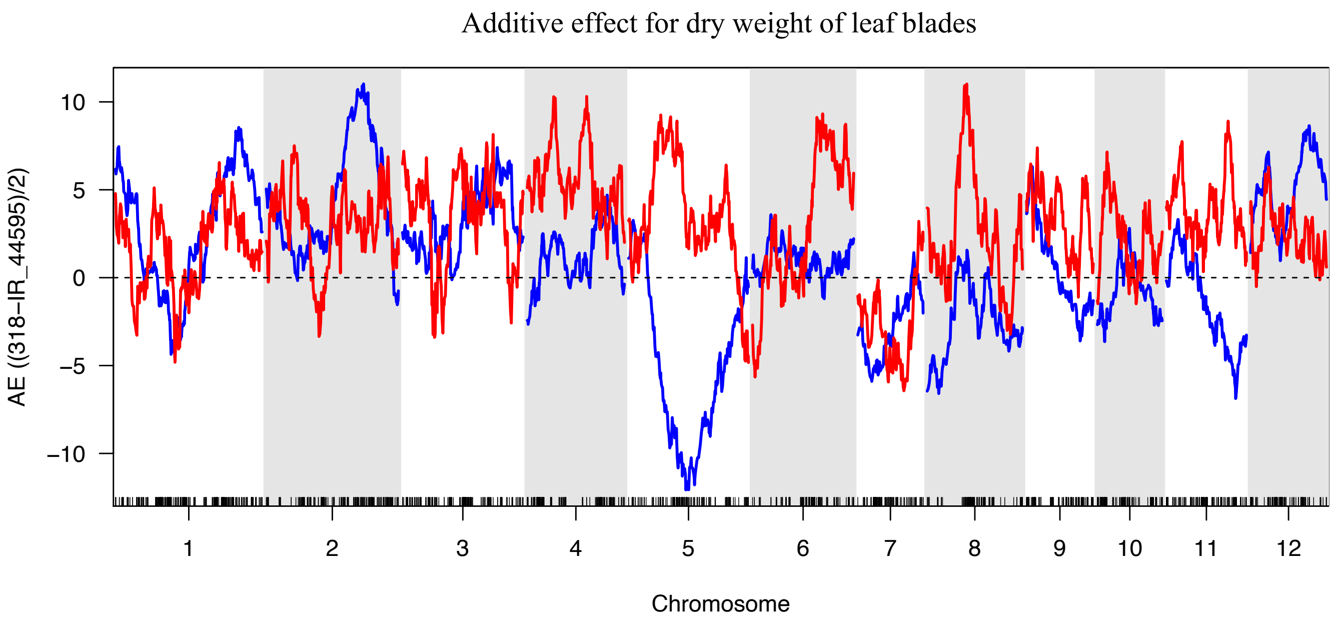
Additive effect for dry weight of leaf blades
AE ((318−IR_44595)/2)
10
5
0
−5
−10
1
2
3
4
5
6
7
8
9
10
11
12
Chromosome

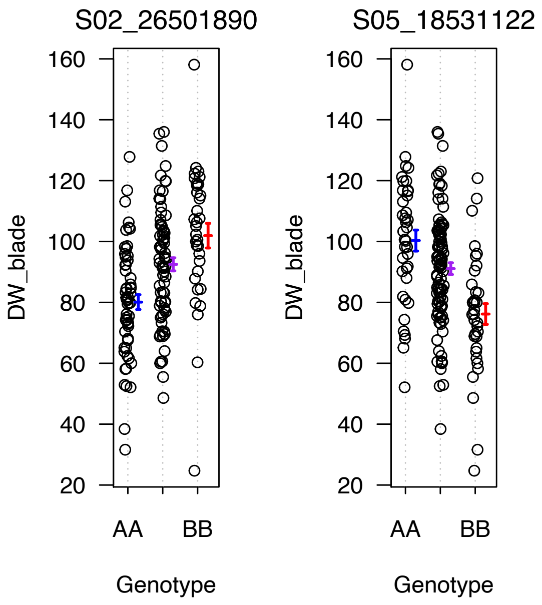
S02_26501890
DW_blade
160
140
120
100
80
60
40
20
AA
BB
Genotype
S05_18531122
DW_blade
160
140
120
100
80
60
40
20
AA
BB
Genotype

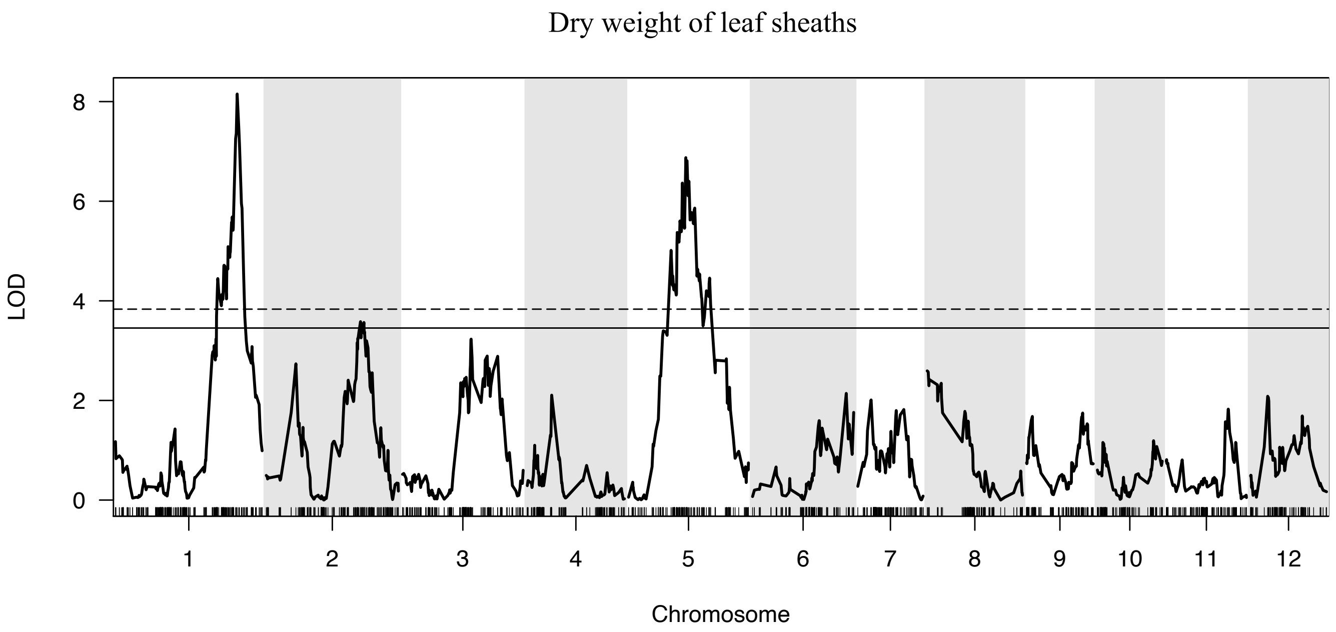
Dry weight of leaf sheaths
LOD
8
6
4
2
0
1
2
3
4
5
6
7
8
9
10
11
12
Chromosome

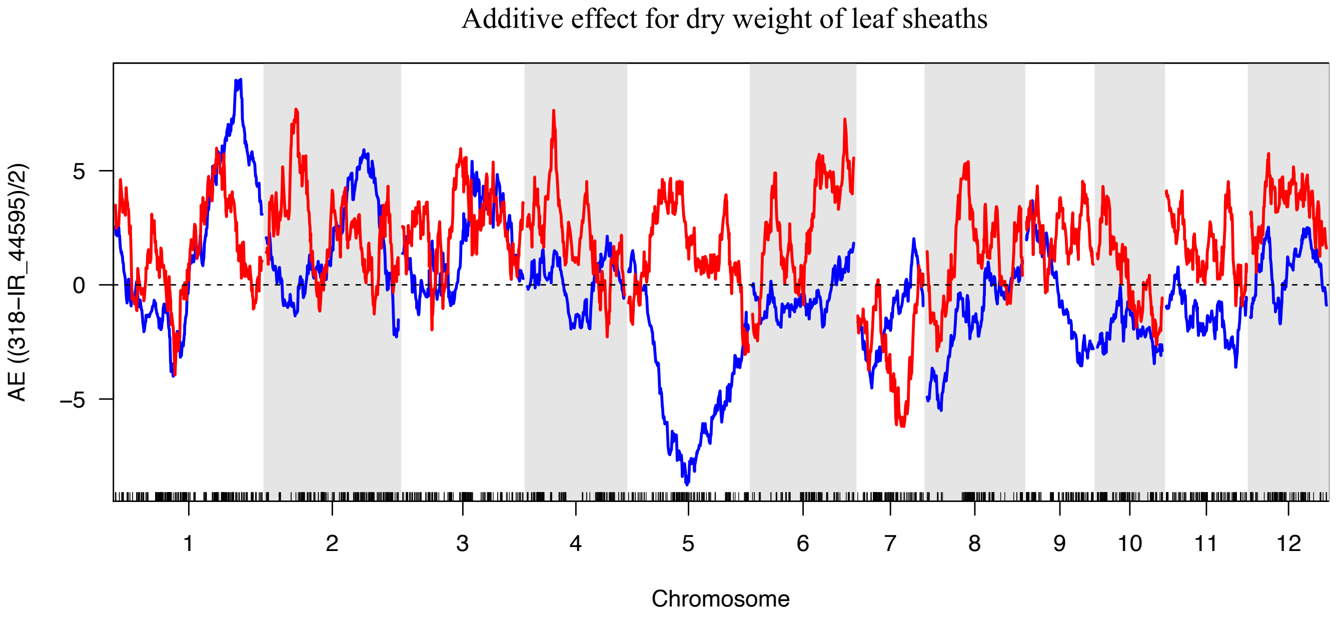
Additive effect for dry weight of leaf sheaths
AE ((318−IR_44595)/2)
5
0
−5
1
2
3
4
5
6
7
8
9
10
11
12
Chromosome

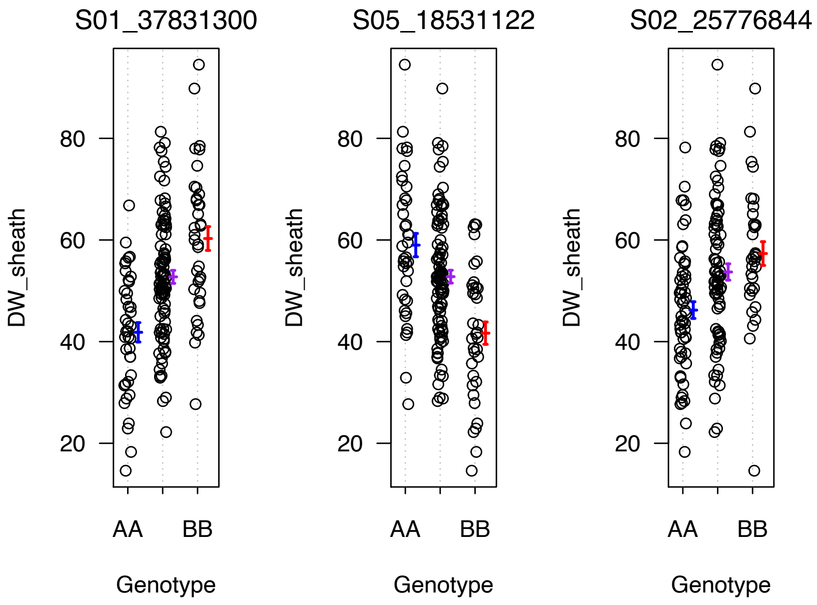
S01_37831300
S05_18531122
S02_25776844
DW_sheath
80
60
40
20
AA
BB
Genotype

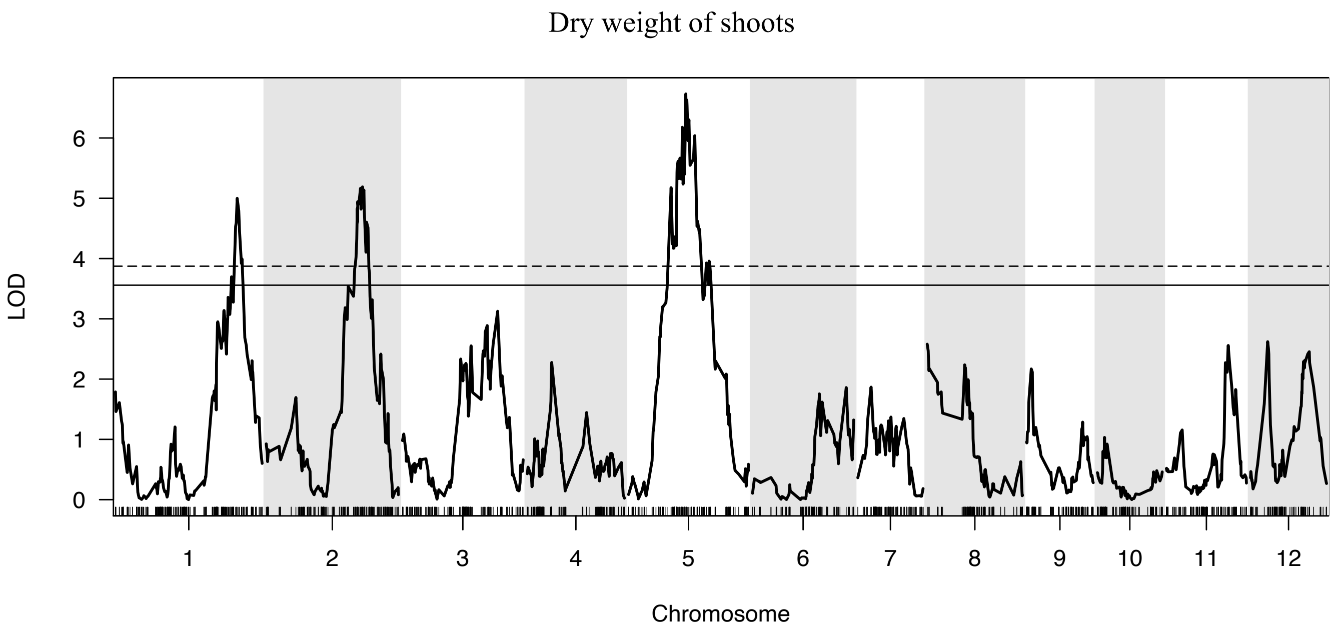
Dry weight of shoots
LOD
0
1
2
3
4
5
6
1 2 3 4 5 6 7 8 9 10 11 12
Chromosome

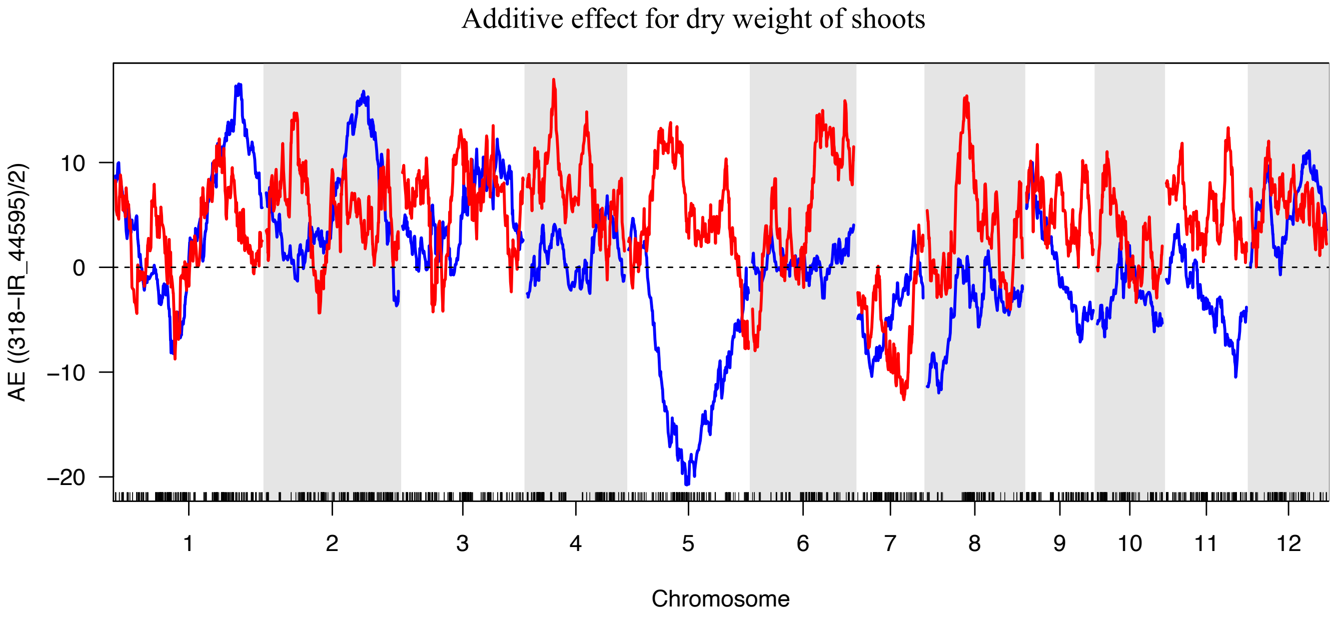
Additive effect for dry weight of shoots
AE ((318−IR_44595)/2)
−20
−10
0
10
1 2 3 4 5 6 7 8 9 10 11 12
Chromosome

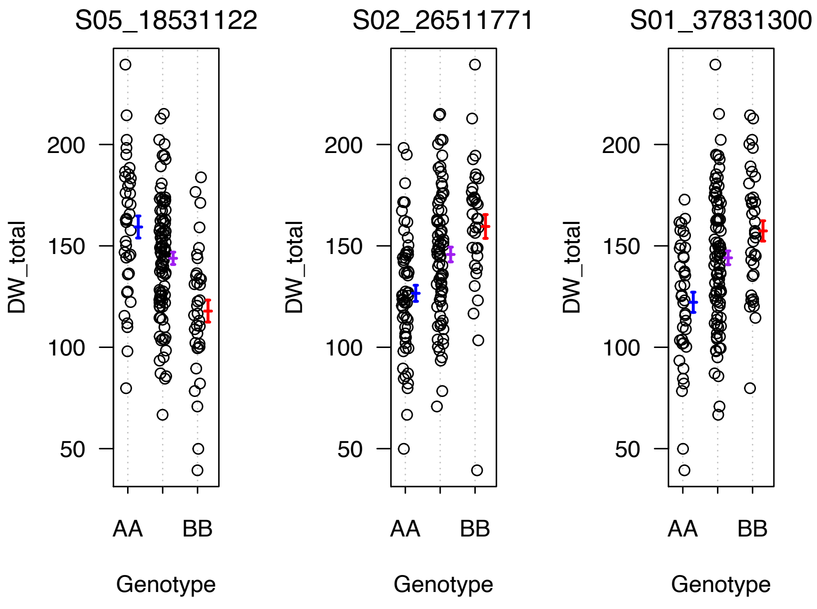
S05_18531122
S02_26511771
S01_37831300
DW_total
50
100
150
200
AA
BB
Genotype
